# Assessing the Impact of the Prostate Cancer Patient Empowerment Program (PC-PEP) on Relationship Satisfaction, Quality of Life, and Support Group Participation: A Randomized Clinical Trial

**DOI:** 10.3390/curroncol31100479

**Published:** 2024-10-21

**Authors:** Cory Burgher, Gabriela Ilie, Ross Mason, Ricardo Rendon, Andrea Kokorovic, Greg Bailly, Nikhilesh Patil, David Bowes, Derek Wilke, Cody MacDonald, Markos Tsirigotis, Calvin Butler, David Bell, Jesse Spooner, Robert David Harold Rutledge

**Affiliations:** 1 Undergraduate Medical Education, Faculty of Medicine, Dalhousie University, Halifax, NS B3H 4R2, Canada; 2 Department of Urology, Faculty of Medicine, Dalhousie University, Halifax, NS B3H 4R2, Canada; 3 Department of Radiation Oncology, Dalhousie University, Halifax, NS B3H 4R2, Canada; 4 Department of Community Health and Epidemiology, Dalhousie University, Halifax, NS B3H 4R2, Canada

**Keywords:** prostate cancer, curative treatment, relationship satisfaction, radical prostatectomy, radiation therapy, emotional well-being, functional well-being, spiritual well-being, social well-being, support group attendance

## Abstract

**Background/Objectives:** The Prostate Cancer Patient Empowerment Program (PC-PEP) is a 6-month, home-based intervention aimed at enhancing mental health in men undergoing curative prostate cancer treatment. This exploratory secondary analysis evaluates PC-PEP’s impact on relationship satisfaction, quality of life, and support group attendance among partnered participants. **Methods:** In a crossover randomized clinical trial ClinicalTrials.gov identifier: NCT03660085) of 128 men aged 50–82 scheduled for curative prostate cancer surgery or radiotherapy, 119 participants in relationships were included. Of these, 59 received the 6-month PC-PEP intervention, while 60 were randomized to a waitlist-control arm, receiving standard care for 6 months before starting PC-PEP. The intervention included daily emails with video instructions on mental and physical health, diet, social support, fitness, stress reduction, and intimacy. Outcomes were assessed using the Dyadic Adjustment Scale (DAS) and the Functional Assessment of Cancer Therapy–Prostate (FACT-P). **Results:** While relationship satisfaction remained stable, a significant improvement in emotional well-being was observed at 12 months in participants undergoing radiation therapy (*p* = 0.045). The PC-PEP intervention also led to significantly higher support group attendance at both 6 months (*p* = 0.001) and 12 months (*p* = 0.003), emphasizing its role in fostering social support and community engagement. **Conclusions:** The PC-PEP program effectively maintains relationship satisfaction and enhances emotional well-being, particularly in patients with fewer physical side effects. Its design promotes comprehensive care by integrating physical, psychological, and social support, making it a valuable resource for improving the quality of life in prostate cancer patients and potentially applicable to other cancer types.

## 1. Introduction

Prostate cancer is the most common cancer among Canadian males, with approximately one in eight men expected to be diagnosed in their lifetime [1]. Although survival rates are relatively high, men living with prostate cancer often endure significant mental health challenges, including anxiety and depression. Research indicates that the prevalence of these mental health issues is higher in men with prostate cancer compared to the general male population, with rates of depression ranging from 15% to 25% and anxiety from 15% to 27% [2,3,4,5]. These psychological challenges are compounded by the side effects associated with prostate cancer treatments, such as urinary incontinence, erectile dysfunction, and bowel dysfunction, which profoundly impact patients’ mental health, quality of life, and relationships [5,6,7]. 

Protective factors, such as strong social support and higher relationship satisfaction, have been identified as crucial for patients undergoing prostate cancer treatment [8,9,10]. However, many patients feel uncomfortable discussing their cancer or its treatment side effects due to a perception that they should be self-reliant or concerned about burdening others [9,11]. This reluctance can lead to social withdrawal, exacerbating the psychological burden of the disease. Moreover, treatment side effects, particularly those affecting sexual function, can strain intimate relationships, a concern expressed by both patients and their partners [6,12].

In response to the need for comprehensive physical and psychological support for prostate cancer patients, the Prostate Cancer Patient Empowerment Program (PC-PEP) was developed. PC-PEP is a 6-month home-based intervention designed to equip patients with skills to improve their mental health, enhance self-efficacy, and mitigate treatment-related side effects, such as urinary and sexual dysfunction, as well as weight gain [13,14,15,16,17,18]. The program has demonstrated success in reducing mental distress and improving oncological outcomes in initial studies. A randomized controlled trial (RCT) investigating the effects of PC-PEP on newly diagnosed prostate cancer patients showed that early exposure to the program significantly improved mental health and reduced the severity of treatment side effects [13]. 

Despite these promising results, the impact of PC-PEP on relationship satisfaction, quality of life indices, and attendance at local support groups has not yet been explored. The PC-PEP program, which was compared against the usual standard of care over a 6-month period, provides an opportunity to assess these important outcomes. This paper presents an exploratory secondary analysis of RCT data to examine the effects of PC-PEP on relationship satisfaction among men who self-reported being in a relationship. Additionally, exploratory analyses will assess the program’s impact on health-related quality of life and participation in community support groups. These outcomes will be examined with respect to the timing of the PC-PEP intervention and the specific prostate cancer treatment modalities used.

## 2. Materials and Methods

### 2.1. Secondary Analysis of a Randomized Clinical Trial

This secondary analysis was conducted using data from the Prostate Cancer Patient Empowerment Program (PC-PEP) trial, a randomized controlled trial with a delayed crossover design. The trial took place in Halifax, Nova Scotia, between December 2019 and January 2021 and aimed to assess the impact of the PC-PEP intervention compared to standard care for men with prostate cancer. The trial was registered with ClinicalTrials.gov under the identifier NCT03660085. A total of 171 men diagnosed with prostate cancer and scheduled for curative treatment were enrolled. All participants provided informed consent, and the trial adhered to the ethical standards of the Declaration of Helsinki, with approval from the Nova Scotia Health Authority (protocol number: 1024822). The trial was reported following the CONSORT guidelines, and detailed information can be found in previously published materials [13]. Participants were recruited through the Urology and Radiation Oncology Departments at the Queen Elizabeth II Health Sciences Centre and by advertisements in prostate cancer support groups. Individuals could also self-refer from the Maritime provinces. Eligibility included a biopsy-confirmed prostate cancer diagnosis, age 18 or older, scheduled for curative treatment within six months, physician clearance for physical activity, and access to digital tools for intervention delivery. Participants needed to be able to read English and willing to attend three study visits over a 12-month period. Of the 171 men screened, 3 were deemed ineligible, and 28 opted out, leaving 140 participants who were randomly assigned to either the intervention or control group. One participant withdrew, and another 11 were excluded for not meeting post-randomization criteria. For this analysis, the sample size was limited to 119 participants who were in a relationship at baseline.

Participants completed online health-related quality-of-life surveys at baseline, 6 months, and 12 months using the REDCap system hosted by Nova Scotia Health. Additional biometric measurements were taken in person, and medical chart reviews were conducted to gather cancer stage, hormone therapy details, treatment modality, and cancer recurrence status. After baseline, participants underwent a physical assessment and were randomly allocated to either the intervention group or the control group. Six months later, the control group began the intervention, while the original intervention group continued with access to the materials.

### 2.2. PC-PEP Intervention

Details of the study protocol are provided in earlier publications [13]. The PC-PEP intervention, lasting 6 months, consisted of daily video content sent via email, promoting health-supporting behaviors. Participants received 3–5 min videos featuring co-authors GI and RDHR, which provided educational content and encouraged physical, mental, and social activities. The videos were supplemented with demonstrations of each activity, organized into a weekly schedule. Participants were encouraged to engage in daily exercise, including twice-weekly resistance training with elastic exercise bands, and to perform pelvic-floor exercises three times daily with optional text reminders. Relaxation techniques were incorporated, using a stress reduction biofeedback device loaned to participants for 6 months. Both the strength and pelvic-floor exercises increased in difficulty throughout the program, tailored to individual fitness levels.

The program also offered guidance on nutrition, emphasizing a diet rich in fruits and vegetables, and addressed lifestyle habits such as sleep hygiene and vitamin D intake. Educational content on intimacy and relationships, particularly related to erectile dysfunction and communication, was also included. Social support strategies were promoted, encouraging participants to build deeper connections with loved ones. Participants had the option of weekly check-ins with co-participants and monthly Zoom sessions with the entire intervention group.

To strengthen relationships and connections, the program provided practical tools for improving communication with loved ones, solutions for erectile dysfunction, and education on intimacy and sexuality. In addition to daily videos, participants were encouraged to attend a monthly online videoconference with the program leads, which featured mini-education sessions on various aspects of the program, breakout room sessions among participants, and larger group discussions. Optional weekly calls with other participants and monthly support group conferences with the PC-PEP team and participants were also available and encouraged.

### 2.3. Dyadic Adjustment Scale (DAS)

Relationship satisfaction was assessed using the Dyadic Adjustment Scale (DAS) [19]. This 32-item questionnaire employs Likert scales to evaluate participants’ perceptions of their romantic relationships. The total score on the DAS can reach a maximum of 151, with a score of 114 representing the norm for a happily married couple, while scores below 97 indicate relationship dissatisfaction. The DAS comprises four subscales that measure specific dimensions of the relationship: (1) satisfaction, (2) Cohesion, (3) Consensus, and (4) Affection. The satisfaction subscale consists of 10 items that assess aspects such as the frequency of quarrels, discussions of separation, and feelings of happiness in the relationship. The Cohesion subscale includes 5 items evaluating the frequency of engaging in shared interests, stimulating exchanges of ideas, laughing together, calm discussions, and collaborating on projects. The Consensus subscale comprises 13 items measuring the level of agreement on family finances, religious matters, household tasks, and life philosophy. Finally, the Affection subscale consists of 4 items that assess agreement on demonstrations of affection, sexual relations, fatigue affecting sex, and expressions of love. The total DAS score is obtained by summing all item scores. The DAS has demonstrated a good internal consistency, with a reported Cronbach’s α of 0.80 for the total DAS score, which is comparable to that reported in the literature [20].

### 2.4. Functional Assessment of Cancer Therapy–Prostate (FACT-P)

Health-related quality of life was assessed using the Functional Assessment of Cancer Therapy–Prostate (FACT-P) questionnaire [21]. This 39-item instrument employs a 5-point Likert scale to measure health-related quality of life over the past 7 days in men with prostate cancer. Three subscales of the FACT-P were evaluated: Social Well-being, Emotional Well-being, and Functional Well-being.

The Social Well-being subscale consists of 7 items assessing aspects such as closeness to friends, emotional support from family, support from friends, family acceptance of illness, communication with family about illness, closeness with a partner, and satisfaction with sex life. The Emotional Well-being subscale comprises 6 items evaluating feelings of sadness, satisfaction with coping with illness, loss of hope, nervousness, worry about dying, and concern about a worsening condition. The Functional Well-being subscale includes 7 items that assess the ability to work, fulfillment from work, ability to enjoy life, acceptance of illness, quality of sleep, enjoyment of hobbies, and contentment with current quality of life.

Responses for each item range from 0 (“Not at all”) to 4 (“Very much”). An overall sense of well-being was obtained by summing the scores of each subscale. This scale demonstrates acceptable internal consistency, with a reported Cronbach’s α of 0.89 for the FACT-P total score, which is comparable to that reported in the literature [22].

### 2.5. Functional Assessment of Chronic Illness Therapy–Spiritual Well-Being (FACIT-Sp-12)

Spiritual well-being was assessed using the Functional Assessment of Chronic Illness Therapy–Spiritual Well-Being scale (FACIT-Sp-12) [23]. This 12-item questionnaire employs a 5-point Likert scale to evaluate spiritual well-being over the past 7 days in adults aged 18 and older living with chronic illness. The FACIT-Sp-12 contains three subscales: Meaning, Peace, and Faith.

The Meaning subscale consists of 4 items that assess perceived reasons for living, the sense of having a productive life, a sense of purpose, and the presence or absence of meaning and purpose. The Peace subscale also includes 4 items evaluating feelings of peace, difficulties in finding peace of mind, the ability to reach deep within oneself for comfort, and a sense of internal harmony. The Faith subscale comprises 4 items that assess comfort and strength derived from faith or spiritual beliefs, the perceived strengthening of faith due to illness, and the belief that things will be okay regardless of the illness’s outcome.

Responses for each item range from 0 (“Not at all”) to 4 (“Very much”). An overall score for spiritual well-being is obtained by summing the scores of each subscale. The FACIT-Sp-12 has demonstrated acceptable internal consistency, with a reported Cronbach’s α of 0.89 for the total score, which is consistent with values reported in the literature [24].

### 2.6. Weekly Adherence to Practicing Different Types of Intimacy with Their Partner

Throughout the 6-month intervention, participants completed weekly online adherence surveys each Sunday, documenting their engagement in various forms of connection and intimacy with their partner over the preceding week (Supplementary Materials). These surveys tracked the frequency (0 to 7 days/week) of daily engagement in at least one form of intimacy. The types of intimacy assessed included emotional intimacy (sharing personal feelings with a partner, being vulnerable, and expressing authentic emotions); intellectual intimacy (exchanging ideas and thoughts, discussing both positive and negative perspectives, and exploring how each person processes issues of importance or interest); physical intimacy (engaging in physical closeness, which could include proximity, touch, kissing, cuddling, hugging, caressing, or sexual activity. This could also involve affectionate interactions with pets); recreational intimacy [participating in shared activities, whether active or passive, such as skating, skiing, playing cards, dancing, golfing, swimming, walking, watching movies, hiking, traveling, or attending classes together (e.g., yoga or exercise classes)]; and self-intimacy (cultivating an awareness of one’s own emotions, demonstrating self-care, and fostering a deep connection with personal thoughts and feelings, alongside practicing self-compassion and love).

These dimensions of intimacy reflect a holistic approach to fostering relational closeness and personal well-being. Although the questions were not part of a validated questionnaire, evidence suggests that engaging in emotional, intellectual, physical, and recreational intimacy can positively influence relationship satisfaction and overall well-being. For instance, studies have highlighted the important role of emotional and physical intimacy in enhancing relationship quality and promoting relational resilience [25].

### 2.7. Sample Characteristics at Baseline

Baseline characteristics included occupation (categorized as occupation code 1 vs. other, coded 0), Charlson Comorbidity Index [26], and non-specific psychological distress as measured by Kessler’s 10 [27], a tool commonly used in research and clinical practice to detect non-specific psychological distress within the past 30 days. Scores of ≥20 indicated the presence of significant distress and the need for clinical treatment (coded 1), while scores <20 indicated the absence of significant distress (coded 0). The Screening for Distress tool [28] was used to identify cancer-related distress among patients over the past 7 days, with results coded as 1 for identified distress and 0 for lack of symptoms. Additionally, support group attendance in the community prostate cancer support group over the past 6 months was assessed at both 6 and 12 months.

### 2.8. Prognostic Covariates

Prognostic covariate data collected at baseline included patient age (in years), treatment modality (coded as 1 for radical prostatectomy and 2 for radiation therapy), and the time elapsed between randomization and treatment onset (in days). These variables have been previously established as significantly impacting health-related quality of life in prostate cancer patients [2,13,29,30].

### 2.9. Sample Size Calculation and Methodological Considerations

The sample size calculation for this RCT was conducted for the primary outcome, focusing on screening positive for non-specific psychological distress, with details reported elsewhere [13]. However, the sample size for the secondary outcomes, such as relationship satisfaction and support group attendance, was not calculated a priori, making this analysis exploratory. The final analysis included 119 participants who were in a relationship at baseline.

Similar studies in the field of prostate cancer psychosocial interventions have utilized comparable sample sizes. For instance, a study which assessed psychological and relational outcomes was conducted with approximately 50 participants per group [31,32]. This suggests that our sample size of 119 participants falls within an appropriate range for detecting medium effect sizes in similar psychosocial outcomes, though we acknowledge the exploratory nature of our secondary analyses.

### 2.10. Statistical Analysis 

Statistical analyses were performed using SPSS version 28. No survey attrition was observed during the PC-PEP trial; however, Dyadic Adjustment Scale (DAS) data were missing for two participants, accounting for less than 1.7% of the sample. The first participant, who was in the radical prostatectomy group, lacked both 6-month and 12-month DAS data due to the loss of a relationship and was excluded from all DAS analyses. The second participant, from the radiation therapy group, was missing DAS data at 6 months due to a similar reason and was excluded from the 6-month analysis.

Baseline demographic characteristics of study participants were summarized with continuous variables presented as means and analyzed using independent *t*-tests or Mann–Whitney U tests, and categorical variables presented as counts and analyzed using Pearson χ^2^ tests. Results of two-level linear modeling analyses (REML estimation), stratified by group and treatment modality, for relationship satisfaction (DAS) and quality of life (FACT-P and FACIT-Sp-12) at baseline, 6 months, and 12 months, were presented comparing the effects of the waitlist control versus the PC-PEP intervention. These analyses were adjusted for covariates; unadjusted results are provided in the Supplementary Materials. All statistical tests were two-sided, with a significance level set at *p* < 0.05.

Intimacy exercises adherence over the 26-week period for the early and late PC-PEP groups were evaluated using generalized linear mixed modeling (GLMM) through the SPSS GENLINMIXED procedure. The model included a random intercept for each participant and a random slope for time, examining the interaction between time and group (early versus late intervention) with both factors entered as fixed effects, using REML estimation. The number of days patients practiced the various forms of intimacy practices recommended was modeled using a binomial distribution with a LOGIT link function. Statistical significance was set at a 2-sided *p*-value of less than 0.05.

## 3. Results

### 3.1. Demographic Measures

The majority of participants were white (95%), identified as cis-gender men (100%), and were either retired or employed (63%). Most participants resided in Nova Scotia (95%) at the time of baseline data collection. All participants self-reported being in a relationship, with the majority being married (88%) or living with their partner (8%), and nearly all were in a heterosexual relationship (99%). Baseline Dyadic Adjustment Scale (DAS) scores averaged 123 in the intervention group and 118 in the control group, indicating overall relationship satisfaction. Statistically significant covariates included the length of the relationship (*p* = 0.06) and the Charlson Comorbidity Index (*p* = 0.016) (see Table 1).

**Figure 1:**
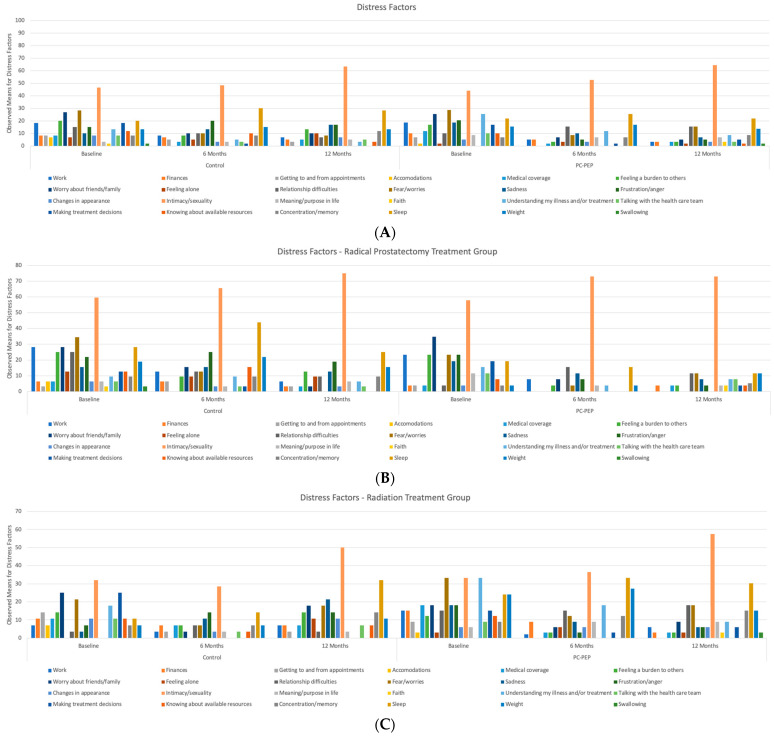
Observed means for (**A**) self-reported distress factors between the control and PC-PEP groups at baseline, 6 months, and 12 months and stratified representations by treatment; (**B**) radical prostatectomy, (**C**) radiation therapy among 119 curative prostate cancer patients treated in Nova Scotia, Canada.

Figure 1 provides an overall representation of the current areas of distress among participants at baseline, 6 months, and 12 months, offering insight into the main concerns that cause distress from the patients’ perspective. It illustrates that, over the past seven days, patients who underwent radical prostatectomy as their primary treatment reported increasing distress related to intimacy and sexuality. In the waitlist control group, distress levels were 46% at baseline, 48% at 6 months, and 62% at 12 months, while in the early PC-PEP group, they were 43% at baseline, 52% at 6 months, and 63% at 12 months. Other distress needs, such as fear/worries and concerns about family and friends, which approached 30% at baseline, showed reductions at both 6 and 12 months in both groups. However, sleep-related distress remained elevated in the waitlist control group, approaching 30% at both 6 and 12 months, while it decreased over time in the early PC-PEP group, with rates of 25% at baseline, 21% at 6 months, and 21% at 12 months. All other distress needs that approached 30% at baseline, including fear and concerns about family and friends, were reduced by 6 to 12 months in both groups.

For patients who underwent radiation therapy as their primary treatment, even higher levels of distress related to intimacy and sexuality were reported compared to the surgery group. In the waitlist control group, distress levels were 60% at baseline, 65% at 6 months, and 75% at 12 months, while in the early PC-PEP group, they were 58% at baseline, 72% at 6 months, and 72% at 12 months. Other distress needs, such as concerns about family and friends, which approached 35% at baseline, showed reductions at both 6 and 12 months in the early PC-PEP group. Additionally, other distress factors that approached 30% at baseline, including fear, sadness, frustration, anger, work-related stress, feelings of being a burden to others, isolation, relationship difficulties, and concerns about family and friends, were reduced by 6 to 12 months in both groups. These results were not subjected to statistical significance testing, as the primary aim was to provide a descriptive overview of the sample characteristics from the patients’ perspective. Given the exploratory nature of this analysis and the large number of categories (over 24) being compared, conducting multiple statistical tests could lead to an increased risk of Type I errors. Additionally, the granular evaluation involved in such a large number of comparisons would likely yield limited interpretive value. Therefore, these findings should be viewed as indicative of general trends rather than definitive statistical outcomes.

### 3.2. Dyadic Adjustment Scale (DAS) Results

A two-level linear model analysis was conducted to compare DAS scores between the waitlist control and PC-PEP intervention groups at the 6-month and 12-month data collection points while controlling for covariates (Table 2). 

**Figure 2:**
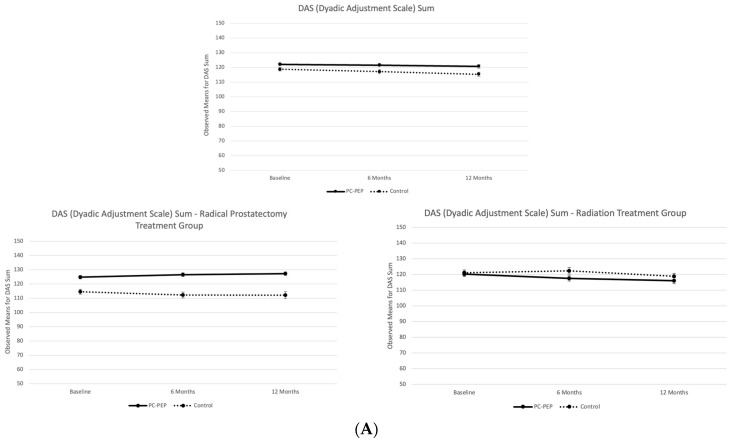

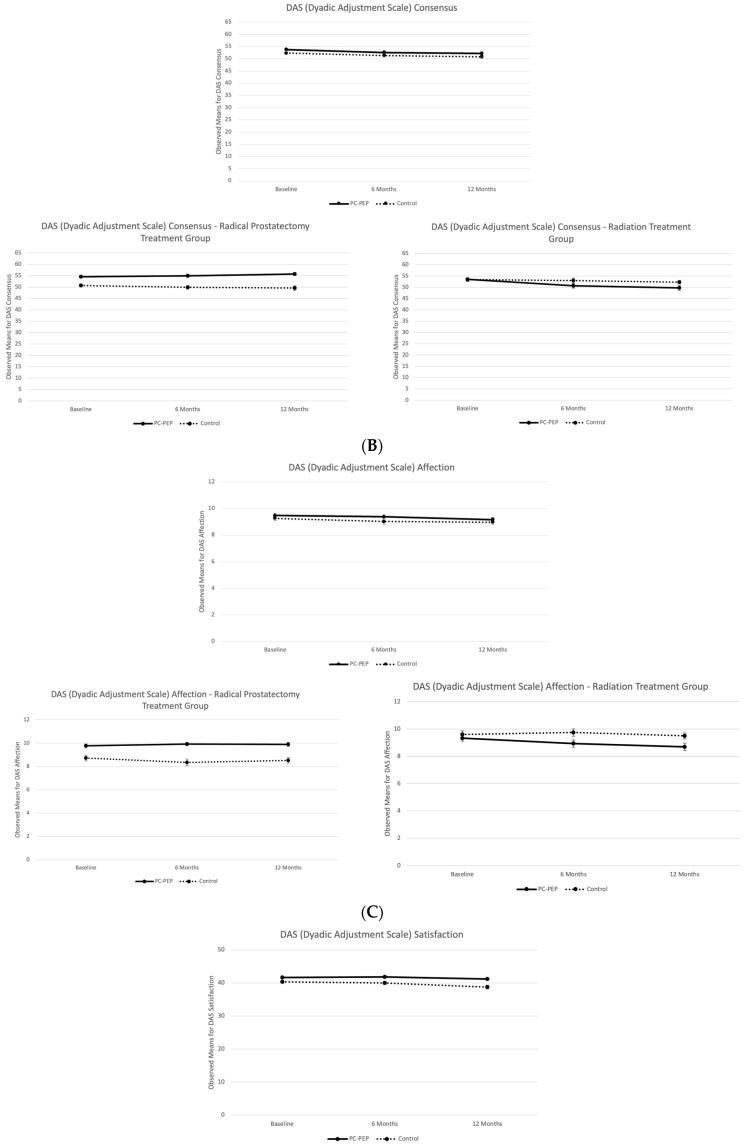

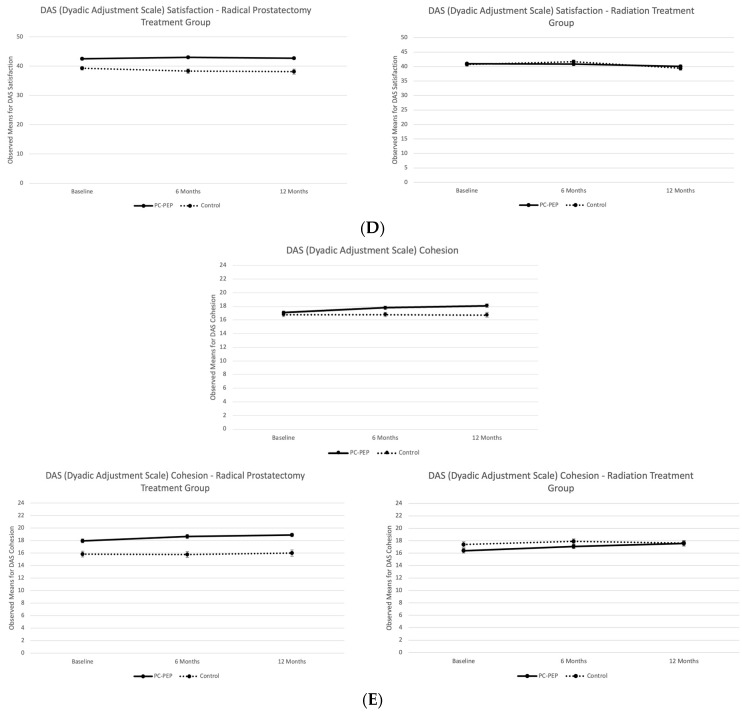
Observed means for (**A**) Dyadic Adjustment Scale (DAS) sum, (**B**) Consensus, (**C**) Affection, (**D**) satisfaction, and (**E**) Cohesion subscales between the control and PC-PEP groups at baseline, 6 months, and 12 months, with stratified representations by treatment among 119 curative prostate cancer patients treated in Nova Scotia, Canada.

Although the PC-PEP group exhibited slightly higher DAS sum scores and subscale scores across all subscales (Figure 2), the group effect was not statistically significant for either the DAS sum scores or any of the DAS subscales at either time point.

Further analyses stratified the data by treatment modality. Among patients undergoing radical prostatectomy, both DAS sum scores and subscale scores were consistently higher in the PC-PEP intervention group across all time points (Figure 2). However, the group effect remained non-significant at each time point (Table 2B). Conversely, among patients undergoing radiation therapy, DAS sum scores, as well as the Consensus and Affection subscales, were slightly higher in the waitlist control group (Figure 2), but again, the group effect was not significant for the DAS or any subscales at either time point (Table 2C).

These findings suggest that the PC-PEP intervention did not have a statistically significant impact on the overall relationship satisfaction of patients who reported being in a current relationship during the trial.

### 3.3. Functional Assessment of Cancer Therapy–Prostate (FACT-P) Results

A two-level linear analysis was conducted to compare FACT-P scores between the waitlist control and PC-PEP intervention groups at the 6-month and 12-month data collection points, controlling for covariates (Table 3A). Statistically significant improvements in emotional well-being were observed among patients in the early PC-PEP group compared to the waitlist control group (*p* = 0.038) from baseline to 12 months. Pairwise comparisons indicated that the increase in emotional well-being was significant among participants in the PC-PEP group [2.2 (95% CI: 1.04 to 3.4), *p* < 0.001], whereas no significant change was observed in the waitlist control group [0.47 (95% CI: −0.67 to 1.6), *p* = 0.4] over the same period. Additionally, emotional well-being scores at 12 months were significantly higher in the early PC-PEP group compared to the waitlist control group [1.8 (95% CI: 0.27 to 3.2), *p* = 0.02].

Subsequent analyses stratified by treatment modality revealed statistically significant changes in emotional well-being (*p* = 0.045) from baseline to 12 months among patients undergoing radiation therapy (Table 3B). Pairwise comparisons showed a significant increase in emotional well-being among participants in the PC-PEP group [estimated mean increase: 2.3 (95% CI: 0.58 to 4.09), *p* = 0.01], while no significant change was observed in the waitlist control group [−0.32 (95% CI: −2.2 to 1.6), *p* = 0.7] over the same period. However, the estimated mean difference in emotional well-being at 12 months between the groups was not statistically significant [1.8 (95% CI: −0.47 to 4.1), *p* = 0.1].

These findings suggest that early PC-PEP intervention may effectively improve emotional well-being over 12 months, with this effect potentially being more pronounced in patients undergoing radiation therapy.

### 3.4. Functional Assessment of Chronic Illness Therapy–Spiritual Well-Being (FACIT-Sp-12)

**Figure 3:**
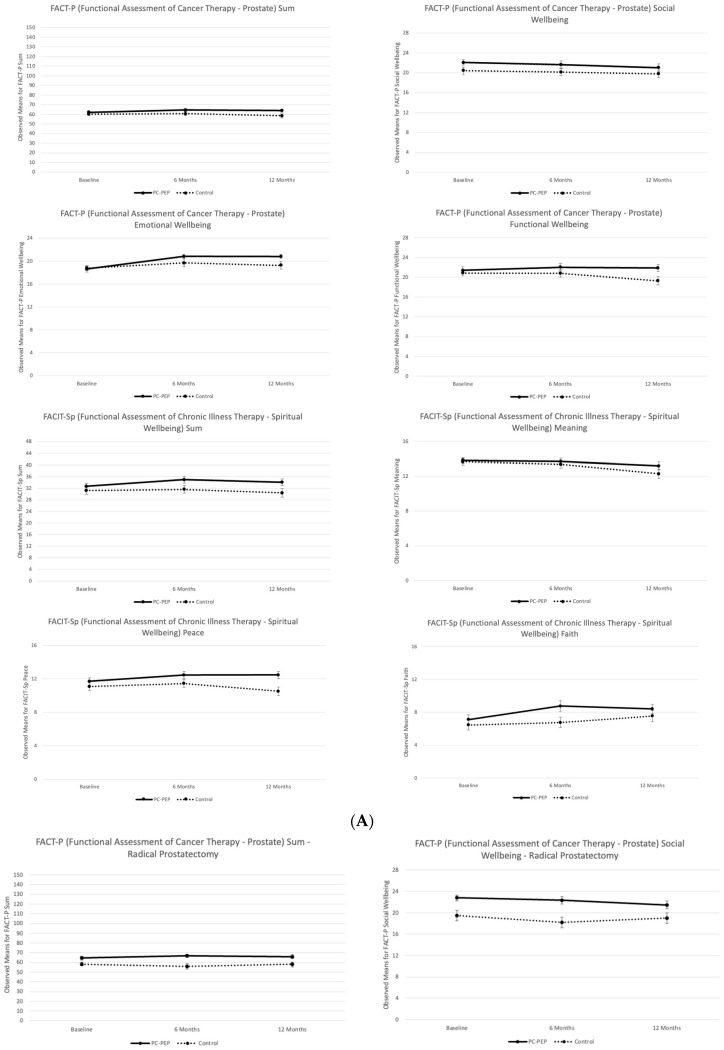

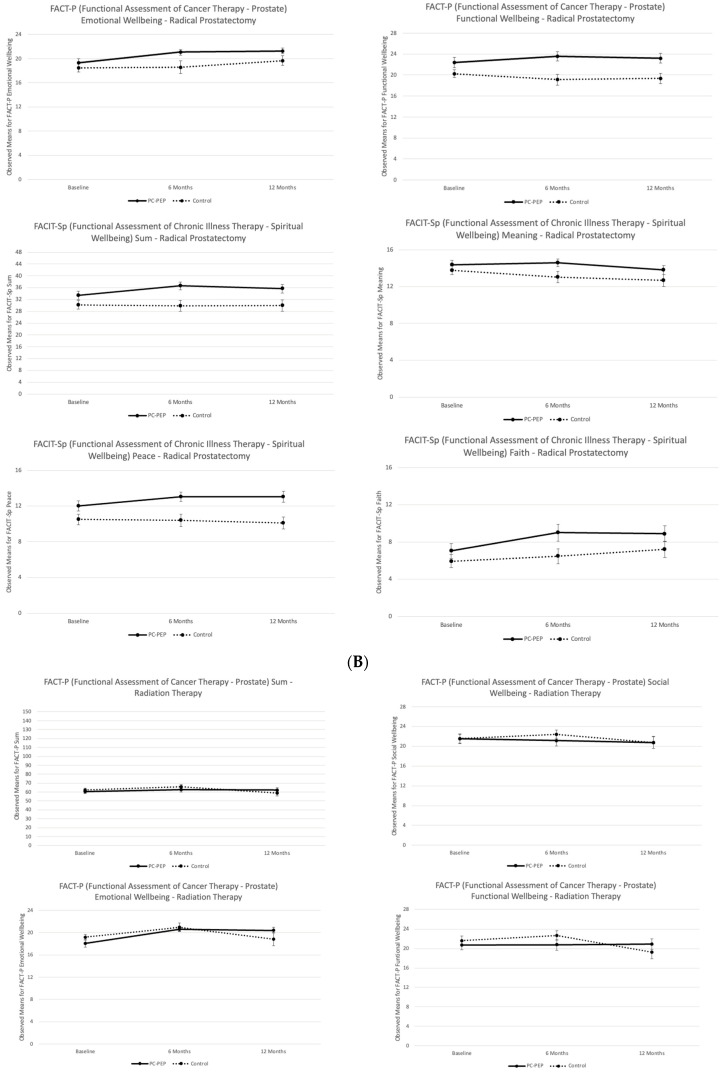

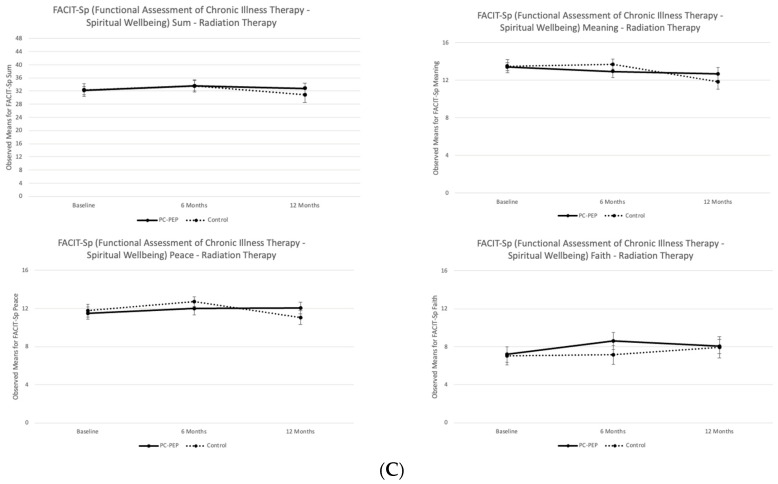
Observed means for (**A**) Functional Assessment of Cancer Therapy–Prostate (FACT-P) Sum and Social Well-being, Emotional Well-being, and Functional Well-being Subscales and Functional Assessment of Chronic Illness Therapy–Spiritual Well-being (FACIT-Sp-12) Sum and Meaning, Peace, and Faith Subscales between the waitlist control and PC-PEP groups at baseline, 6 months, and 12 months and stratified representations by treatment (**B**) radical prostatectomy vs. (**C**) radiation among 119 curative prostate cancer patients treated in Nova Scotia, Canada.

A two-level linear analysis was conducted to compare FACIT-Sp-12 scores between the waitlist control and PC-PEP intervention groups at the 6-month and 12-month data collection points while controlling for covariates (Table 3A). Participants in the early PC-PEP group reported statistically significantly higher peace scores (*p* = 0.037) on the FACIT-Sp-12 from baseline to 12 months compared to the waitlist control group. However, pairwise comparisons did not show significant changes in peace scores from baseline to 12 months within either the waitlist control group [−0.55 (95% CI: −1.4 to 0.30), *p* = 0.2] or the PC-PEP group [0.74 (95% CI: −0.12 to 1.6), *p* = 0.093]. Despite this, peace scores were significantly higher in the PC-PEP group at 12 months, with a notable mean difference between the groups [2.1 (95% CI: 0.84 to 3.4), *p* = 0.001] (Figure 3).

Subsequent analyses stratified by treatment group revealed statistically significant changes in FACIT-Sp-12 sum scores (*p* = 0.048) from baseline to 6 months among participants undergoing radical prostatectomy (Table 3B). Pairwise comparisons indicated a significant increase in spiritual well-being from baseline to 6 months in the early PC-PEP group [3.0 (95% CI: 0.53 to 5.5), *p* = 0.019], along with a significant mean difference between the waitlist control and PC-PEP groups at 6 months [6.6 (95% CI: 2.2 to 11), *p* = 0.004]. However, these differences in FACIT-Sp-12 sum scores were no longer significant at 12 months (*p* = 0.2). Within the radical prostatectomy group, a statistically significant difference in participants’ experienced inner peace was observed from baseline to 12 months (*p* = 0.042). Although pairwise comparisons did not show significant changes in peace from baseline to 12 months within either the waitlist control group [-0.41 (95% CI: −1.3 to 0.46) *p* = 0.4] or the PC-PEP group [0.95 (95% CI: −0.028 to 1.9), *p* = 0.06], a significant mean difference was observed at 12 months, with the early PC-PEP group reporting higher levels of peace compared to the waitlist control group [2.9 (95% CI: 1.2 to 4.7), *p* = 0.001]. No significant differences in FACIT-Sp-12 or its subscales were observed among participants undergoing radiation therapy (Table 3C).

These results suggest that early PC-PEP intervention may enhance overall feelings of peace after 12 months, particularly among prostate cancer patients undergoing radical prostatectomy.

### 3.5. Adherence to Intimacy Exercises Results

**Figure 4:**
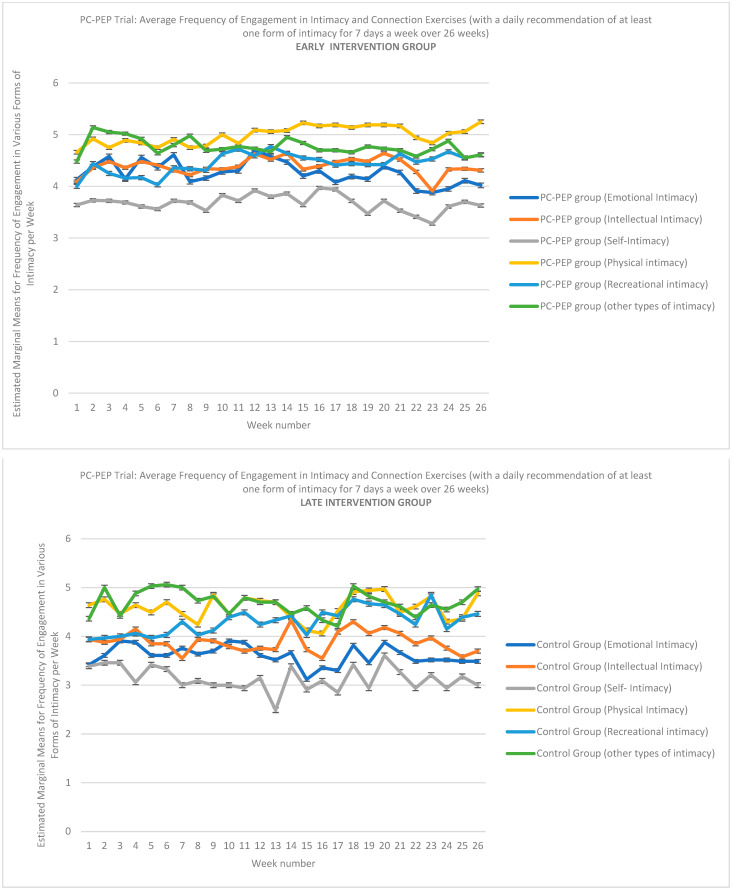
The observed means for adherence to the intimacy and connection components of the PC-PEP program were recorded for both the intervention group (who began the intervention early) and the waitlist control group (who started the intervention 6 months after the trial began) over a 26-week period. These observations included the total number of times per day in each week patients spent engaging in the various forms of intimacy prescribed by the program. These data were collected from 128 prostate cancer patients undergoing curative treatment in Nova Scotia, Canada, who participated in the PC-PEP Trial. Note: PC-PEP = Prostate Cancer–Patient Empowerment Program.

The PC-PEP trial evaluated the engagement of participants in various forms of intimacy and connection exercises, with a daily recommendation of at least one form of intimacy each day over 26 weeks (Supplementary Materials). The early intervention group, who began the program at the start of the trial, showed relatively consistent engagement across different intimacy types (see Figure 4). The estimated marginal means for emotional intimacy, intellectual intimacy, self-intimacy, physical intimacy, recreational intimacy, and other forms of intimacy were tracked weekly. Emotional and physical intimacy, in particular, showed higher levels of consistent engagement, averaging between 4 to 5 days per week, with occasional fluctuations. In contrast, self-intimacy and recreational intimacy were performed less frequently, typically around 3 to 4 days per week.

For the late intervention (control) group, who started the program 6 months post-trial, engagement in the various forms of intimacy followed similar patterns (Figure 4). However, their overall adherence levels were somewhat lower, with emotional and physical intimacy showing less frequent engagement than the early group, averaging closer to 4 days per week. Intellectual intimacy, recreational intimacy, and other types of intimacy displayed similar trends, with fluctuations around 3 to 4 days per week.

Generalized linear mixed modeling (GLMM) was used to analyze intimacy and connection adherence between the early and late groups over a 26-week period. No significant interaction between group and time was observed (see Table 4), suggesting that both early and late exposure to the program led to similar sustained engagement in the intimacy and connection exercises. The GLMM analysis also revealed a main effect of time for both physical and recreational intimacy engagement (see Table 4). As shown in Table 5, over time, there was an increased likelihood of participants engaging in physical intimacy (β = 0.038, t = 2.85, *p* = 0.006, OR = 1.04, 95% CI: 1.01 to 1.07) and recreational intimacy (β = 0.028, t = 3.00, *p* = 0.004, OR = 1.03, 95% CI: 1.01 to 1.05), regardless of group randomization.

### 3.6. Support Group Attendance Results

A statistically significant increase in community-based prostate cancer support group attendance was observed in both the waitlist control and early PC-PEP groups from baseline to 6 months [mean increase: 0.37 (95% CI: 0.25 to 0.49), *p* < 0.001 and 0.17 (95% CI: 0.048 to 0.29), *p* = 0.006, respectively]. The mean difference between the groups at 6 months was also statistically significant [0.20 (95% CI: 0.076 to 0.31), *p* = 0.001], indicating that participants in the early PC-PEP group had higher support group attendance compared to the waitlist control group.

A statistically significant increase in support group attendance was also observed from baseline to 12 months, with a greater increase seen in the PC-PEP group compared to the waitlist control group [0.41 (95% CI: 0.28 to 0.53), *p* < 0.001 and 0.22 (95% CI: 0.094 to 0.34), *p* < 0.001, respectively]. The mean difference between groups at 12 months was statistically significant [0.19 (95% CI: 0.067 to 0.32), *p* = 0.003], indicating that participants in the PC-PEP group continued to have higher support group attendance at this time point.

When stratified by treatment group, no significant differences in support group attendance were observed from baseline to 6 months or baseline to 12 months among participants undergoing radical prostatectomy. However, within the radiation therapy group, while there was no significant difference from baseline to 6 months, a statistically significant increase in support group attendance was observed from baseline to 12 months among participants in the PC-PEP group [0.39 (95% CI: 0.23 to 0.56), *p* < 0.001]. The mean difference between the PC-PEP and waitlist control groups at 12 months was also significant [0.23 (95% CI: 0.054 to 0.40), *p* = 0.011], indicating higher support group attendance among participants in the PC-PEP group undergoing radiation therapy.

## 4. Discussion

In this secondary analysis of the Prostate Cancer Patient Empowerment Program (PC-PEP), we assessed the effects of a 6-month, home-based patient empowerment program on relationship satisfaction, patient well-being, and support group attendance among men undergoing curative prostate cancer treatment. The findings revealed significant improvements in emotional well-being, peace, and support group attendance for participants in the early PC-PEP intervention group compared to the waitlist control group.

### 4.1. Interpretation of the Dyadic Adjustment Scale (DAS) Results

The analysis did not show significant changes in Dyadic Adjustment Scale (DAS) sum scores or any of its subscales, suggesting that the PC-PEP intervention did not significantly impact relationship satisfaction, regardless of the timing of the intervention. This outcome is likely influenced by the high baseline levels of relationship satisfaction in both the waitlist control and PC-PEP groups (118 and 122 out of a possible 151 points, respectively). High baseline levels of relationship satisfaction can create a ceiling effect, making it challenging to detect further improvements [33]. This phenomenon is common in psychological interventions, where participants with high initial scores often show less improvement due to limited room for gains.

Despite the lack of significant changes in DAS scores, participants demonstrated excellent adherence to the prescribed intimacy exercises, with both early and late intervention groups exceeding the recommended exercise goals. This high level of engagement suggests that the structured intimacy activities were well received by participants and may have played a role in maintaining the high levels of relationship satisfaction observed throughout the study. These results build on prior evidence showing the mental health benefits of PC-PEP in men undergoing prostate cancer treatment [13,18].

Additionally, the longstanding nature of many participants’ relationships (mean of 37 years in the waitlist control group and 31 years in the PC-PEP group) likely contributed to the results. Long-term relationships tend to develop stable interaction patterns, particularly in communication and intimacy, which can be resistant to change, especially over the relatively short duration of clinical trials [34]. Interventions aimed at improving relationship satisfaction may require further extended periods to show significant effects.

Nevertheless, maintaining high relationship satisfaction is a positive outcome, especially in the context of chronic illness, where stressors can easily strain relationships. The education and strategies provided in the PC-PEP program may have helped participants sustain their high levels of relationship satisfaction, which is strongly associated with better mental health, improved quality of life, and reduced mortality [35]. High relationship satisfaction is consistently linked to various positive health outcomes, emphasizing the importance of maintaining strong, supportive relationships during significant life stressors, such as a prostate cancer diagnosis [36,37].

### 4.2. Interpretation of the Functional Assessment of Cancer Therapy–Prostate (FACT-P) Results 

While improvements in various components of the FACT-P were observed among participants in the early PC-PEP group, only the emotional well-being subscale at 12 months reached statistical significance. When stratified by treatment group, this statistically significant improvement remained only in the radiation therapy group. This discrepancy may be explained by the lower burden of treatment-associated side effects experienced by the radiation group compared to those undergoing radical prostatectomy. For example, less severe urinary incontinence and erectile dysfunction are often seen in patients undergoing radiation therapy, which may contribute to higher emotional well-being [38].

Indeed, intimacy and sexuality emerged as more prominent self-reported distress factors at 12 months among participants undergoing radical prostatectomy compared to those undergoing radiation therapy. These findings align with previous research indicating that treatment-related side effects, such as urinary incontinence and erectile dysfunction, can significantly impact emotional well-being, particularly in patients who undergo more invasive treatments like surgery [39,40]. The significant improvement in emotional well-being among the radiation group suggests that the PC-PEP intervention may be particularly effective for patients experiencing fewer physical side effects, allowing them to focus more on the psychosocial aspects of recovery [41].

These results are consistent with earlier findings from the same study, which showed that self-efficacy and specific illness perceptions played a critical role in mediating the effects of the PC-PEP intervention on reducing psychological distress [16]. The intervention significantly improved self-efficacy and illness perceptions, such as personal control and emotional response, which in turn contributed to reduced psychological distress [13]. This highlights the importance of psychological empowerment in improving emotional well-being among prostate cancer patients.

The integration of physical and psychological support through the PC-PEP program may enhance overall well-being, reinforcing the importance of a comprehensive approach to cancer care that addresses both the physical and emotional needs of patients.

### 4.3. Interpretation of the Functional Assessment of Chronic Illness Therapy–Spiritual Well-Being (FACIT-Sp-12) Results

Significant improvements were observed only after 12 months in the peace subscale, both in the whole-sample analysis and among participants undergoing radical prostatectomy in the stratified analysis. However, the increase in peace scores from baseline to 12 months was not significant for either the waitlist control or PC-PEP groups when analyzed separately. Instead, the PC-PEP group had significantly higher peace scores at 12 months compared to the waitlist control group, suggesting that while the intervention did not markedly increase spiritual well-being, it did help maintain a higher level of inner peace over time.

This finding is consistent with previous studies that emphasize the role of structured interventions in maintaining psychological stability among cancer patients. For instance, studies on mindfulness-based stress reduction (MBSR) programs have similarly shown that these interventions may not drastically alter spiritual well-being scores but can help sustain or slightly improve existing levels of inner peace and emotional regulation [42]. The lack of significant changes in faith-related distress in the PC-PEP group could reflect the participants’ focus on more immediate concerns related to their physical health and emotional well-being, as suggested by the low baseline rates of faith-related distress. Previous research has shown that cancer patients often prioritize interventions that directly address their physical and psychological symptoms, while spiritual concerns might take a secondary role unless they are particularly salient to the individual [43]. 

### 4.4. Interpretation of the Support Group Attendance Results

The Prostate Cancer–Patient Empowerment Program (PC-PEP) was designed not only as a structured intervention for improving physical and psychological health but also as a means to foster community engagement and build lasting support networks among participants. The program included optional weekly connections with two other co-participants, mentorship opportunities, and monthly video conferences where participants could interact with each other. These components were crucial in facilitating meaningful connections, encouraging participants to actively engage with their peers, and offering a platform for shared experiences and mutual support.

The most consistent improvements observed in the study were in support group attendance, where participants in the early intervention PC-PEP group demonstrated higher attendance rates than those in the waitlist control group at both the 6- and 12-month time points. Even after both groups had received the PC-PEP intervention at 12 months, the early intervention group continued to show greater participation in support groups. This suggests that early exposure to the PC-PEP may enhance patients’ understanding of the importance of psychological support and encourage ongoing engagement in such activities.

This increased engagement can be attributed to the program’s emphasis on human connection and interaction. By encouraging participants to connect with peers, mentors, and healthcare professionals, PC-PEP helps bridge the gap between community-based support and the resources offered by the medical system. This bridging is crucial as it empowers patients to take an active role in their own care, utilizing both medical and community resources to enhance their overall well-being [16]. Research supports the idea that such integrative approaches, which combine structured medical interventions with community support, are essential for comprehensive patient care, particularly in chronic illnesses like cancer [9,44]. 

The program’s design, which includes regular one-on-one interactions and group activities, helps patients feel more connected and supported, reducing feelings of isolation that are common during cancer treatment. This sense of community not only helps in overcoming psychological challenges but also encourages patients to remain engaged with community-based activities long after their treatment ends. Such sustained engagement is critical for maintaining the psychological and emotional gains made during the intervention.

Furthermore, by linking patients with both their immediate peers and broader support networks, PC-PEP fosters a sense of belonging and empowerment, which can lead to improved self-efficacy and mental health outcomes. The program’s success in increasing support group attendance highlights the importance of a comprehensive, human-centered approach to patient care—one that integrates personal connections, community involvement, and structured medical support. This approach ensures that patients are not only treated for their physical ailments but are also supported in building the psychological resilience and social networks necessary for long-term health and well-being [16,45].

In conclusion, PC-PEP’s ability to connect patients with community resources and encourage active participation in their own care underscores the importance of interactive, human-centered programs in healthcare. By bridging the gap between the medical system and community support, PC-PEP provides a model for comprehensive care that can significantly enhance the quality of life for prostate cancer patients during and after treatment. Focusing on improving support group attendance through programs such as PC-PEP or other community resources may improve the quality of life for prostate cancer patients. As advancements in medical therapies have extended the course of prostate cancer treatments and survivorship, it is vital to address the often unmet care needs that accompany these long-term treatments. Despite the availability of support, many patients are hesitant to engage in help-seeking behaviors [46]. Previous research has demonstrated the positive effects of group-based support on individuals living with chronic illness, including improved self-care, self-efficacy, and quality of life [47]. This study further demonstrates that the PC-PEP intervention equips prostate cancer patients with the knowledge about the importance of support and empowers them to access and adhere to support groups. These findings supplement previous results, which indicated that the PC-PEP intervention effectively activates the role of the patient in their own care and decreases mental distress among prostate cancer patients [13,16]. 

### 4.5. Limitations

This secondary analysis has several limitations that must be considered. Firstly, the participant sample was predominantly composed of white, high socioeconomic status, cis-gendered men in heterosexual marriages. This demographic homogeneity limits the generalizability of the findings to more diverse populations. The experiences and outcomes observed in this study may not reflect those of individuals from different racial, socioeconomic, or cultural backgrounds or those with varying sexual orientations and gender identities.

Secondly, while the sample size calculation was thoroughly conducted for the primary outcomes, such as mental distress, it was not performed for the secondary outcomes evaluated in this analysis, such as relationship satisfaction and support group attendance. This is why the analysis presented here is exploratory in nature. The lack of a priori power calculations for these secondary outcomes means that the study might be underpowered to detect smaller yet clinically meaningful differences in these outcomes.

Moreover, the study relied heavily on self-reported data collected through questionnaires. The subjective nature of self-reported data introduces the possibility of personal biases and inconsistencies in interpretation, which may affect the reliability of the findings. Participants’ responses could be influenced by social desirability bias or recall bias, leading to either overestimation or underestimation of their true experiences.

The questionnaires used in this study also have inherent limitations. For example, the Dyadic Adjustment Scale (DAS), which measures relationship satisfaction at a single point in time, may not capture the dynamic and evolving nature of relationships. Relationships fluctuate over time, and a one-time measurement may fail to account for these changes, limiting the ability to draw definitive conclusions about long-term relationship satisfaction.

Moreover, the DAS, Functional Assessment of Cancer Therapy–Prostate (FACT-P), and Functional Assessment of Chronic Illness Therapy–Spiritual Well-Being (FACIT-Sp-12) scales were developed based on traditional Western cultural norms. As a result, these instruments may not accurately reflect the experiences of individuals from non-Western cultures or those who do not conform to conventional Western relationship structures. Cultural differences in perceptions of health, spirituality, and relationships may lead to misinterpretation or underrepresentation of certain experiences, potentially skewing the results.

Finally, while the FACT-P provides valuable insights into the quality-of-life issues faced by prostate cancer patients, its reliance on self-reported measures means that the results may be unintentionally influenced by the presence of other comorbid conditions. Patients with additional health issues may report lower quality of life scores due to the combined effects of multiple conditions, making it challenging to isolate the impact of prostate cancer and its treatment on their well-being.

These limitations highlight the need for future studies to include more diverse participant samples, consider longitudinal designs to capture the evolving nature of relationships, and incorporate culturally sensitive measurement tools. Expanding the scope of research in these ways will help to ensure that the findings are more broadly applicable and accurately reflect the experiences of all prostate cancer patients.

## 5. Conclusions

This secondary analysis highlights the significant impact of the Prostate Cancer–Patient Empowerment Program (PC-PEP) on improving emotional well-being and support group attendance among men diagnosed with prostate cancer. The PC-PEP program, developed with direct patient engagement, represents a comprehensive approach to patient care that addresses not only the physical but also the psychological challenges associated with prostate cancer treatment.

The PC-PEP program is a six-month intervention that integrates daily aerobic and strength training, pelvic floor exercises (kegels) to support urinary and sexual function, dietary changes aimed at preventing prostate cancer progression, and stress reduction techniques utilizing a biofeedback device. In addition to these physical health components, the program emphasizes social and emotional support, with participants encouraged to engage in weekly interactions with co-participants, mentorship opportunities, and monthly video conferences with program leads—a prostate cancer oncologist and a scientist specializing in prostate cancer quality of life research. These elements foster a sense of community and provide ongoing guidance and encouragement, which are crucial for maintaining long-term engagement and emotional resilience.

The results of the study demonstrate that early participation in the PC-PEP program significantly reduces mental distress compared to standard care. Participants who received the intervention immediately after their diagnosis showed a marked decrease in psychological distress, highlighting the importance of timely intervention in improving mental health outcomes. These findings are particularly relevant for clinical care, as they suggest that integrating PC-PEP into standard care protocols can enhance the overall quality of life for prostate cancer patients.

The program’s design, which promotes both physical health and psychological well-being through structured yet flexible activities, makes it an ideal candidate for broader implementation. The ongoing phase 4 implementation trial across Canada and internationally, including in New Zealand, aims to expand the reach of PC-PEP and evaluate its effectiveness in diverse patient populations. This expansion is crucial, as it will provide further evidence of the program’s utility in different cultural and healthcare contexts, paving the way for PC-PEP to become a standard component of prostate cancer care globally.

Moreover, the success of PC-PEP in reducing psychological distress and increasing support group attendance underscores the program’s potential for application in other types of cancer. By focusing on patient empowerment and engagement, PC-PEP offers a model for holistic cancer care that can be adapted and expanded to meet the needs of a broader range of patients. The program’s cost-effectiveness and high patient adherence further support its feasibility for widespread adoption.

In conclusion, the PC-PEP program is a valuable resource that not only addresses the immediate needs of prostate cancer patients but also provides a framework for long-term care that integrates physical, psychological, and social support. As the program continues to expand nationally and internationally, it holds the promise of transforming cancer care by empowering patients to take an active role in their health and well-being, ultimately improving outcomes and quality of life across a range of cancer types.

## Figures and Tables

**Table 1:** Baseline sample characteristics comparing the waitlist control and Prostate Cancer Patient Empowerment Program (PC-PEP) groups among 119 patients undergoing curative prostate cancer treatment in Halifax, Nova Scotia.

	Waitlist Control	PC-PEP	*p* Value
	n = 60	n = 59	
Age (years)	60, 67 (51, 82)	59, 65 (50, 78)	0.11
Age of partner (years)	60, 65 (49, 81)	59, 61 (41, 78)	0.005
Length of relationship (years)	60, 37 (1, 58)	59, 31 (2, 55)	0.031
*Relationship status*			0.16
Married	55, 92%	49, 83%	
Living with a partner/Dating/Not sure how to answer	5, 8%	10, 17%	
Ethnicity, white	60, 98%	54, 91.5%	0.089
Working full/part-time vs. retired or unemployed	22, 37%	21, 36%	0.9
Total household income >$30,000	52, 87%	49, 83%	0.6
*Occupation*			
Specialized middle management vs. other	17, 28%	21, 36%	0.4
Senior management vs. other	14, 23%	18, 31%	0.4
Business/finance/administration vs. other	7, 12%	16, 27%	0.033
Telecommunication/line cable workers vs. other	4, 6.7%	2, 3.4%	0.4
Police officer vs. other	2, 3.3%	1, 1.7%	0.6
Insurance/real estate/financial brokerage manager vs. other	3, 5%	1, 1.7%	0.3
Firefighter vs. other	1, 1.7%	1, 1.7%	1
Administrative and related vs. other	17, 28%	17, 29%	0.9
Natural resources vs. other	10, 17%	7, 12%	0.5
Metal processing/construction vs. other	8, 13%	4, 6.8%	0.3
Transportation and related vs. other	6, 10%	9, 15%	0.4
Protective services vs. other	8, 13%	9, 15%	0.8
Health/personal care vs. other	4, 6.7%	3, 5.1%	0.7
None of the above vs. other	16, 27%	19, 32%	0.5
Charlson Comorbidity Index (age-adjusted)	60, 2.7 (1, 4)	59, 2.3 (1, 4)	0.017
Time between randomization and active treatment (days)	60, 70 (3, 173)	59, 65 (8, 138)	0.5
*Treatment modality*			
Surgery	32, 53%	26, 44%	0.3
Radiation	28, 47%	33, 56%	
*Diagnosis and treatment-relevant characteristics*			
Stage of cancer			
Risk Category (RP ^1^ + primary RT ^2^ ± HT ^3^) ^4^			0.7
Low	0	0	
Intermediate	16, 52%	14, 56%	
High	15, 48%	11, 44%	
Prescribed ADT ^5^	21, 35%	25,43%	0.4
Dyadic Adjustment Scale Sum	60, 118 (53, 145)	59, 122 (52, 147)	0.3
Dyadic Adjustment subscale (Consensus)	60, 52 (29, 65)	59, 54 (25, 65)	0.3
Dyadic Adjustment subscale (Affection)	60, 9.1 (0, 12)	59, 9.5 (1, 12)	0.7
Dyadic Adjustment subscale (Satisfaction)	60, 40 (19, 49)	59, 42 (22, 49)	0.11
Dyadic Adjustment subscale (Cohesion)	60, 17 (4, 24)	59, 17 (4, 24)	0.87
K10 ^6^, screening positive for psychological distress and need for clinical treatment	10, 17%	8, 14%	0.6
*Distress Factors*			
Work/school vs. all else	11, 18%	11, 19%	1
Finances vs. all else	5, 8.3%	6, 10%	0.7
Getting to and from appointments vs. all else	5, 8.3%	4, 6.8%	0.8
Accommodations vs. all else	4, 6.7%	1, 1.7%	0.18
Medical coverage vs. all else	5, 8.3%	7, 12%	0.5
Feeling a burden to others vs. all else	12, 20%	10, 17%	0.7
Worry about friends/family vs. all else	16, 27%	15, 25%	0.9
Feeling alone vs. all else	4, 6.7%	1, 1.7%	0.18
Relationship difficulties vs. all else	9, 15%	6, 10%	0.4
Fear/worries vs. all else	17, 28%	17, 29%	0.9
Sadness vs. all else	6, 10%	11, 19%	0.18
Frustration/anger vs. all else	9, 15%	12, 20%	0.5
Changes in appearance vs. all else	5, 8.3%	3, 5.1%	0.5
Intimacy/sexuality vs. all else	28, 47%	26, 44%	0.8
Meaning/purpose in life vs. all else	2, 3.3%	5, 8.5%	0.2
Faith vs. all else	1, 1.7%	0, 0%	0.3
Understanding my illness and/or treatment vs. all else	8, 13%	15, 25%	0.095
Talking with the healthcare team vs. all else	5, 8.3%	6, 10%	0.7
Making treatment decisions vs. all else	11, 18%	10, 17%	0.8
Knowing about available resources vs. all else	7, 12%	6, 10%	0.8
Concentration/memory vs. all else	5, 8.3%	4, 6.8%	0.8
Sleep vs. all else	12, 20%	13, 22%	0.8
Weight vs. all else	8, 13%	9, 15%	0.8
Swallowing vs. all else	1, 1.7%	0, 0%	0.3
Changes in who I am vs. all else	8, 13%	6, 10%	0.6
Other vs. all else	2, 3.3%	2, 3.4%	1
None of the above vs. all else	11, 18%	9, 15%	0.7
Number of distress factors	60, 3.7 (1, 15)	59, 3.9 (1, 13)	0.6
Overall psychological distress in the past week	60, 3.2 (0, 8)	59, 2.9 (0, 9)	0.5
FACT-P ^7^ Sum Well-being	60, 60 (35, 79)	59, 62 (21, 78)	0.3
FACT-P Social Well-being subscale	60, 20 (3, 28)	59, 22 (5, 28)	0.1
FACT-P Emotional Well-being subscale	60, 19 (4, 24)	59, 19 (6, 24)	0.9
FACT-P Functional Well-being subscale	60, 21 (8, 28)	59, 22 (6, 28)	0.3
FACIT-Sp-12 ^8^ Sum Well-being	60, 32 (0, 46)	59, 33 (13, 47)	0.4
FACIT-Sp-12 Meaning subscale	60, 14 (0, 16)	59, 14 (7, 16)	0.9
FACIT-Sp-12 Peace subscale	60, 11 (0, 16)	59, 12 (2, 16)	0.3
FACIT-Sp-12 Faith subscale	60, 6.5 (0, 16)	59, 7.3 (0, 16)	0.3
Sexually active in previous 3 months	46, 77%	43, 73%	0.6
Techniques used to improve sex life			
None vs. some	31, 52%	34, 58%	0.5
Penile injection therapy vs. none or other	0, 0%	1, 1.7%	0.3
Viagra vs. none or other	14, 23%	13, 22%	0.9
Sex toys vs. none or other	4, 6.7%	1, 1.7%	0.18
Erection-independent sexual activities vs. none or other	5, 8.3%	3, 5.1%	0.5
Support group attendance at baseline or ever	1, 1.7%	1, 1.7%	1

Note: Summary statistics are presented as n, mean (min, max) for continuous variables and n (%) for categorical variables, except for PSA, for which n, median (quartiles) are reported. ^1^ Radical prostatectomy; ^2^ Radiation therapy; ^3^ Hormone therapy; ^4^ National Comprehensive Cancer Network (NCCN); ^5^ ADT—Androgen deprivation therapy; ^6^ Kessler’s 10–Assessment of Non-Specific Psychological Distress and Need for Clinical Treatment; ^7^ FACT-P–Functional Assessment of Cancer Therapy–Prostate Cancer Symptom Index. ^8^ FACIT-Sp-12–Functional Assessment of Cancer Therapy–Spiritual Well-being.

**Table 2:** Two-level linear model analysis for the whole sample (**A**) and by treatment modality (**B**,**C**) fitting the Dyadic Adjustment Scale sum score and its subscales (Affection, Consensus, Satisfaction, Cohesion) among 119 prostate cancer patients evaluating differences between groups (waitlist control vs. PC-PEP) from baseline to 6 months, and baseline to 12 months.

*A. Full Sample (n = 119)*	Baseline to 6 Months				6 months to 12 Months			
Level	Parameter Estimate	95% Confidence Interval		*p*	Parameter Estimate	95% Confidence Interval		*p*
		Lower	Upper			Lower	Upper	
**DAS Sum score**								
Group (Control vs. PC-PEP)	−6.3	−14	1.02	0.091	−6.9	−14	0.55	0.069
Time	0.3004	−2.6	3.2	0.8	1.3	−3.7	6.2	0.6
Time × Group (PC-PEP)	1.2	−2.9	5.3	0.6	1.6	−5.4	8.7	0.7
**Consensus score**								
Group (Control vs. PC-PEP)	−2.1	−5.5	1.3	0.2	−2.0	−5.5	1.4	0.3
Time	1.2	−0.75	3.2	0.2	1.6	−1.2	4.3	0.3
Time × Group (PC-PEP)	−0.34	−3.1	2.4	0.8	−0.29	−4.1	3.6	0.9
**Affection score**								
Group (Control vs. PC-PEP)	−0.59	−1.6	0.39	0.2	−0.40	−1.3	0.54	0.4
Time	0.11	−0.39	0.60	0.7	0.29	−0.34	0.91	0.4
Time × Group (PC-PEP)	0.11	−0.59	0.81	0.8	−0.062	−0.95	0.82	0.9
**Satisfaction score**								
Group (Control vs. PC-PEP)	−2.2	−4.6	0.097	0.060	−2.8	−5.2	−0.44	0.021
Time	−0.26	−1.5	1.0	0.7	0.44	−1.1	2.0	0.6
Time × Group (PC-PEP)	0.57	−1.2	2.3	0.5	0.97	−1.2	3.2	0.4
**Cohesion score**								
Group (Control vs. PC-PEP)	−1.2	−2.8	0.49	0.17	-1.6	-3.2	0.11	0.067
Time	−0.74	−1.5	0.040	0.063	−1.0	−2.1	0.026	0.056
Time × Group (PC-PEP)	0.70	−0.40	1.8	0.2	0.96	−0.53	2.5	0.2
***B. Radical Prostatectomy (n = 57)***								
**DAS Sum score**								
Group (Control vs. PC-PEP)	−16	−25	−5.9	0.002	−16	−26	−5.9	0.002
Time	−2.2	−7.1	2.7	0.3	−2.5	−9.9	4.9	0.5
Time × Group (PC-PEP)	6.1	−0.53	13	0.070	6.1	−3.8	16	0.2
**Consensus score**								
Group (Control vs. PC-PEP)	−5.5	−9.3	−1.7	0.006	−6.5	−11	−2.3	0.003
Time	−0.48	−2.7	1.7	0.7	−1.3	−4.8	2.3	0.5
Time × Group (PC-PEP)	1.9	−1.1	4.8	0.2	2.8	−1.9	7.5	0.24
**Affection score**								
Group (Control vs. PC-PEP)	−1.7	−3.0	−0.42	0.010	−1.5	−2.7	−0.30	0.015
Time	−0.17	−0.95	0.61	0.7	−0.16	−1.1	0.77	0.7
Time × Group (PC-PEP)	0.72	−0.33	1.8	0.17	0.50	−0.75	1.8	0.4
**Satisfaction score**								
Group (Control vs. PC-PEP)	−5.1	−8.4	−1.8	0.003	−4.9	−8.4	−1.4	0.007
Time	−0.68	−2.8	1.5	0.5	−0.19	−2.8	2.5	0.9
Time × Group (PC-PEP)	2.1	−0.76	5.0	0.15	1.7	−1.8	5.3	0.3
**Cohesion score**								
Group (Control vs. PC-PEP)	−3.2	−5.5	−0.77	0.010	−3.1	−5.5	−0.71	0.012
Time	−0.80	−1.9	0.28	0.14	−0.87	−2.3	0.54	0.2
Time × Group (PC-PEP)	1.2	−0.26	2.7	0.11	0.99	−0.90	2.9	0.3
***C. Radiation Therapy (n = 61)***								
**DAS Sum score**								
Group (Control vs. PC-PEP)	0.067	−10	10	1.0	−0.54	−11	9.9	0.9
Time	2.3	−1.1	5.7	0.18	4.2	−2.7	11	0.2
Time × Group (PC-PEP)	−3.4	−8.5	1.6	0.17	−1.9	−12	8.2	0.7
**Consensus**								
Group (Control vs. PC-PEP)	−0.36	−5.7	4.9	0.9	0.97	−4.3	6.3	0.7
Time	2.6	−0.56	5.8	0.10	3.8	−0.29	7.9	0.068
Time × Group (PC-PEP)	−2.2	−6.8	2.5	0.4	−2.7	−8.7	3.4	0.4
**Affection score**								
Group (Control vs. PC-PEP)	0.21	−1.2	1.6	0.8	0.43	−0.99	1.9	0.6
Time	0.33	−0.32	0.98	0.3	0.64	−0.24	1.5	0.2
Time × Group (PC-PEP)	−0.47	−1.4	0.48	0.3	−0.53	−1.8	0.76	0.4
**Satisfaction score**								
Group (Control vs. PC-PEP)	−0.11	−3.2	3.0	1	−1.5	−4.7	1.7	0.3
Time	0.11	−1.3	1.5	0.9	0.94	−0.96	2.8	0.3
Time × Group (PC-PEP)	−1.1	−3.2	1.0	0.3	0.35	−2.5	3.2	0.8
**Cohesion score**								
Group (Control vs. PC-PEP)	0.42	−1.9	2.7	0.7	−0.40	−2.8	2.0	0.7
Time	−0.68	−1.8	0.46	0.2	−1.2	−2.7	0.44	0.2
Time × Group (PC-PEP)	0.18	−1.5	1.9	0.8	0.94	−1.4	3.3	0.4

Note: The following baseline covariates were included in the model: age, treatment modality, and the number of days elapsed between trial randomization and the start of active treatment.

**Table 3:** Two-level linear model analysis for the whole sample (**A**) and by treatment modality (**B**,**C**) fitting the Functional Assessment of Cancer Therapy–Prostate (FACT-P) and the Functional Assessment of Chronic Illness Therapy–Spiritual Well-being (FACIT-Sp-12) among 119 prostate cancer patients evaluating differences between groups (waitlist control vs. PC-PEP from baseline to 6 months, and baseline to 12 months.

*A. Full Sample (N = 119)*	Baseline to 6 Months				6 Months to 12 Months			
Level	Parameter Estimate	95% Confidence Interval		*p*	Parameter Estimate	95% Confidence Interval		*p*
		Lower	Upper			Lower	Upper	
	**FACT-P Sum Score**							
Group (Control vs. PC-PEP)	−4.2	−8.7	0.30	0.067	−5.7	−10	−1.1	0.016
Time	−2.3	−4.9	0.33	0.086	−1.6	−5.0	1.7	0.3
Time × Group (PC-PEP)	1.8	−1.9	5.4	0.34	3.4	−1.3	8.1	0.15
	**FACT-P Social Well-being Score**							
Group (Control vs. PC-PEP)	−1.6	−3.5	0.32	0.10	−1.3	−3.3	0.68	0.2
Time	0.46	−0.60	1.5	0.39	1.0	−0.41	2.4	0.16
Time × Group (PC-PEP)	−0.19	−1.7	1.3	0.8	−0.37	−2.4	1.6	0.7
	**FACT-P Emotional Well-being Score**							
Group (Control vs. PC-PEP)	−1.2	−2.7	0.19	0.090	−1.8	−3.2	−0.27	0.020
Time	−2.2	−3.4	−1.1	<0.001	−2.2	−3.4	−1.0	<0.001
Time × Group (PC-PEP)	1.4	−0.27	3.0	0.10	1.7	0.10	3.4	0.038
	**FACT-P Functional Well-being Score**							
Group (Control vs. PC-PEP)	−1.4	−3.4	0.60	0.17	−2.7	−4.7	−0.63	0.011
Time	−0.50	−1.7	0.71	0.4	−0.46	−1.9	1.0	0.5
Time × Group (PC-PEP)	0.62	−1.1	2.3	0.5	2.0	−0.026	4.1	0.053
	**FACIT-Sp-12 Sum Score**							
Group (Control vs. PC-PEP)	−3.7	−7.0	−0.42	0.028	−4.2	−7.6	−0.70	0.019
Time	−2.1	−3.9	−0.34	0.020	−1.4	−3.9	1.1	0.3
Time × Group (PC-PEP)	1.7	−0.79	4.2	0.18	2.2	−1.3	5.7	0.2
	**FACIT-Sp-12 Meaning Score**							
Group (Control vs. PC-PEP)	−0.54	−1.6	0.55	0.3	−1.1	−2.3	0.10	0.071
Time	0.20	−0.50	0.90	0.6	0.66	−0.28	1.6	0.17
Time × Group (PC-PEP)	0.096	−0.89	1.1	0.9	0.70	−0.62	2.0	0.3
	**FACIT-Sp-12 Peace Score**							
Group (Control vs. PC-PEP)	−1.1	−2.4	0.11	0.074	−2.1	−3.4	−0.84	0.001
Time	−0.70	−1.4	0.027	0.059	−0.74	−1.6	0.12	0.093
Time × Group (PC-PEP)	0.32	−0.71	1.4	0.5	1.3	0.078	2.5	0.037
	**FACIT-Sp-12 Faith Score**							
Group (Control vs. PC-PEP)	−2.0	−3.8	−0.28	0.023	−0.93	−2.7	0.83	0.3
Time	−1.6	−2.6	−0.71	<0.001	−1.3	−2.5	−0.093	0.035
Time × Group (PC-PEP)	1.3	0.00060	2.6	0.050	0.18	−1.5	1.9	0.8
***B. Radical Prostatectomy (n = 57)***								
	**FACT-P Sum Score**							
Group (Control vs. PC-PEP)	−11	−17	−5.1	<0.001	−8.0	−14	−2.3	0.007
Time	−2.3	−6.5	2.0	0.3	−1.4	−5.5	2.6	0.5
Time × Group (PC-PEP)	4.6	−1.0	10	0.11	1.8	−3.7	7.2	0.5
	**FACT-P Social Well-being Score**							
Group (Control vs. PC-PEP)	−4.1	−6.5	−1.6	0.002	−2.5	−4.9	−0.064	0.044
Time	0.60	−0.95	2.2	0.4	1.4	−0.35	3.1	0.12
Time × Group (PC-PEP)	0.71	−1.4	2.8	0.5	−0.82	−3.1	1.5	0.5
	**FACT-P Emotional Well-being Score**							
Group (Control vs. PC-PEP)	−2.6	−4.8	−0.34	0.024	−1.7	−3.7	0.30	0.095
Time	−1.8	−3.7	0.093	0.062	−2.0	−3.5	−0.51	0.009
Time × Group (PC-PEP)	1.7	−0.80	4.3	0.2	0.85	−1.2	2.8	0.4
	**FACT-P Functional Well-being Score**							
Group (Control vs. PC-PEP)	−4.4	−6.9	−1.8	0.001	−3.8	−6.4	−1.2	0.004
Time	−1.1	−3.1	0.98	0.3	−0.79	−2.7	1.1	0.4
Time × Group (PC-PEP)	2.2	−0.56	5.0	0.12	1.7	−0.86	4.3	0.2
	**FACIT-Sp-12 Sum Score**							
Group (Control vs. PC-PEP)	−6.6	−11	−2.2	0.004	−5.8	−10	−1.2	0.014
Time	−3.0	−5.5	−0.53	0.019	−2.2	−5.3	0.91	0.16
Time × Group (PC-PEP)	3.4	0.029	6.7	0.048	2.5	−1.7	6.6	0.2
	**FACIT-Sp-12 Meaning Score**							
Group (Control vs. PC-PEP)	−1.5	−2.9	−0.13	0.033	−1.2	−2.7	0.36	0.13
Time	−0.15	−1.2	0.85	0.8	0.60	−0.51	1.7	0.3
Time × Group (PC-PEP)	0.90	−0.44	2.3	0.18	0.53	−0.96	2.0	0.5
	**FACIT-Sp-12 Peace Score**							
Group (Control vs. PC-PEP)	−2.6	−4.3	−0.95	0.002	−2.9	−4.7	−1.2	0.001
Time	−0.98	−2.1	0.14	0.086	−0.95	−1.9	0.028	0.057
Time × Group (PC-PEP)	1.1	−0.40	2.6	0.15	1.4	0.049	2.7	0.042
	**FACIT-Sp-12 Faith Score**							
Group (Control vs. PC-PEP)	−2.5	−4.8	−0.18	0.035	−1.7	−4.0	0.61	0.15
Time	−1.9	−3.3	−0.57	0.006	−1.9	−3.7	0.016	0.052
Time × Group (PC-PEP)	1.4	−0.42	3.2	0.13	0.57	−1.9	3.1	0.7
***C. Radiation Therapy (n = 61)***								
	**FACT-P Sum Score**							
Group (Control vs. PC-PEP)	2.2	−4.4	9.0	0.5	−3.7	−11	3.7	0.3
Time	−2.2	−5.4	0.90	0.16	−1.8	−7.0	3.5	0.5
Time × Group (PC-PEP)	−1.5	−6.2	3.1	0.5	5.2	−2.6	13	0.19
	**FACT-P Social Well-being Score**							
Group (Control vs. PC-PEP)	0.76	−2.1	3.7	0.6	−0.12	−3.3	3.0	0.9
Time	0.36	−1.1	1.8	0.6	0.76	−1.5	3.0	0.5
Time × Group (PC-PEP)	−1.3	−3.4	0.84	0.2	−0.0076	−3.3	3.3	1.0
	**FACT-P Emotional Well-being Score**							
Group (Control vs. PC-PEP)	0.17	−1.7	2.0	0.9	−1.8	−4.1	0.47	0.12
Time	−2.6	−4.0	−1.1	<0.001	−2.3	−4.1	−0.58	0.010
Time × Group (PC-PEP)	0.72	−1.4	2.8	0.5	2.7	0.059	5.3	0.045
	**FACT-P Functional Well-being Score**							
Group (Control vs. PC-PEP)	1.3	−1.7	4.3	0.4	−1.8	−5.0	1.4	0.3
Time	−0.061	−1.5	1.4	0.9	−0.18	−2.4	2.0	0.9
Time × Group (PC-PEP)	−0.98	−3.1	1.1	0.4	2.5	−0.78	5.8	0.13
	**FACIT-Sp-12 Sum Score**							
Group (Control vs. PC-PEP)	−1.6	−6.4	3.2	0.5	−2.9	−8.2	2.3	0.3
Time	−1.4	−4.0	1.2	0.3	−0.67	−4.6	3.2	0.7
Time × Group (PC-PEP)	0.11	−3.7	3.9	1.0	2.1	−3.6	7.9	0.5
	**FACIT-Sp-12 Meaning Score**							
Group (Control vs. PC-PEP)	0.18	−1.5	1.8	0.8	−1.3	−3.2	0.64	0.19
Time	0.49	−0.50	1.5	0.3	0.73	−0.78	2.2	0.3
Time × Group (PC-PEP)	−0.70	−2.2	0.76	0.3	0.92	−1.3	3.1	0.4
	**FACIT-Sp-12 Peace Score**							
Group (Control vs. PC-PEP)	0.024	−1.8	1.8	1.0	−1.6	−3.5	0.35	0.11
Time	−0.49	−1.5	0.49	0.3	−0.55	−1.9	0.85	0.4
Time × Group (PC-PEP)	−0.48	−1.9	0.96	0.5	1.3	−0.79	3.3	0.2
	**FACIT-Sp-12 Faith Score**							
Group (Control vs. PC-PEP)	−1.8	−4.6	0.92	0.19	−0.10	−2.8	2.6	0.9
Time	−1.4	−2.7	−0.098	0.035	−0.85	−2.4	0.73	0.3
Time × Group (PC-PEP)	1.3	−0.63	3.2	0.18	−0.044	−2.4	2.3	1.0

Note: The following baseline covariates are included in the model: age, treatment modality, and the number of days elapsed between trial randomization and start of active treatment.

**Table 4:** Generalized linear mixed model (GLMM) analysis of the Group (late vs. early PC-PEP delivery) × Time (26 weeks) interaction, evaluating adherence to intimacy and connection engagement recommendations among 128 prostate cancer patients in Halifax, Nova Scotia, Canada.

*Tests of Fixed Effects*			
*Effect*	*df*	*F*	*p-Value*
Emotional intimacy engagement—Average number of days per week			
Time	1.68	0.40	0.53
Group	1.75	2.36	0.13
Group × Time	1.68	0.14	0.71
Intelectual intimacy engagement—Average number of days per week			
Time	1.74	1.74	0.19
Group	1.75	1.21	0.28
Group × Time	1.74	0.84	0.36
Physical intimacy engagement—Average number of days per week			
Time	1.63	6.53	**0.013**
Group	1.83	1.25	0.27
Group × Time	1.63	2.57	0.11
Physical intimacy engagement—Average number of days per week ^1^			
Time	1.69	8.13	**0.006**
Group	1.89	2.23	0.14
Recreational intimacy engagement—Average number of days per week			
Time	1.97	7.00	**0.01**
Group	1.93	0.93	0.34
Group × Time	1.97	0.33	0.57
Recreational intimacy engagement—Average number of days per week ^1^			
Time	1.82	8.98	**0.004**
Group	1.96	1.30	0.26
Self intimacy engagement—Average number of days per week			
Time	1.60	0.17	0.68
Group	1.86	0.55	0.46
Group × Time	1.60	1.95	0.17
Other types of intimacy engagement—Average number of days per week			
Time	1.75	0.11	0.74
Group	1.91	0.72	0.40
Group × Time	1.75	0.012	0.91

^1^ Analysis displays follow-up analyses for significant main effects in the absence of a significant interaction.

**Table 5:** Follow-up PC-PEP intervention adherence analyses evaluating significant Group (late vs. early PC-PEP delivery) × Time (26 weeks) interactions or main effects (where the interaction was found to be not significant) among 128 prostate cancer patients from Halifax, Nova Scotia, Canada.

	β	t	*p*	OR (95% CI)
Physical intimacy engagement—Average number of days per week				
Time	0.038	2.85	0.006	1.04 (1.01–1.07)
Recreational intimacy engagement—Average number of days per week				
Time	0.028	3.00	0.004	1.03 (1.01–1.05)

## Data Availability

Data from this study are available to researchers through a data access process in compliance with patient privacy and protection research acts (NSHA Research Ethics Board).

## Supplementary Materials

The following supporting information can be downloaded at: https://www.mdpi.com/article/10.3390/curroncol31100479/s1, Table S1: Two-level linear model analysis for the whole sample (A) and by treatment modality (B) fitting the Dyadic Adjustment Scale sum score and its subscales (Affection, Consensus, Satisfaction, Cohesion) among 119 prostate cancer patients evaluating differences between groups (waitlist control vs. PC-PEP) from baseline to 6 months and baseline to 12 months *without covariate adjustment*; Table S2: Two-level linear model analysis for the whole sample (A) and by treatment modality (B) fitting the Functional Assessment of Cancer Therapy–Prostate (FACT-P) and the Functional Assessment of Chronic Illness Therapy–Spiritual Well-being (FACIT-Sp-12) among 119 prostate cancer patients evaluating differences between groups (waitlist control vs. PC-PEP from baseline to 6 months, and baseline to 12 months, *without covariate adjustment.*
