# Supplementary material for: Assessing the Impact of the Prostate Cancer Patient Empowerment Program (PC-PEP) on Relationship Satisfaction, Quality of Life, and Support Group Participation: A Randomized Clinical Trial
Source: Curr Oncol. 2024 Oct 21;31(10):6445–74. doi: 10.3390/curroncol31100479 (PMC11506086; doi:10.3390/curroncol31100479)
Supplement: Supplementary file 1 [file curroncol-31-00479-s001.zip › curroncol-3221463-supplementary.pdf]

Weekly compliance surveys PC-PEP RCT:

Compliance

Thank you for your participation in the PC-PEP (Prostate Cancer - Patient Empowerment Program)!

Please complete the survey below.

Thank you!

The following questions are about your engagement in AEROBIC EXERCISES:

During the PC-PEP Intervention/Training with our registered Certified Exercise Practitioner Jeff Zahavich, we encouraged you to do aerobic exercises (walking or substitute) for 90-150 minutes a week.

HOW MANY DAYS THIS WEEK, did you do the AEROBIC EXERCISES that you were prescribed by the Study's Exercise Expert?

- ◯ not at all
- ◯ 1 day
- ◯ 2 days
- ◯ 3 days
- ◯ 4 days
- ◯ 5 days
- ◯ 6 days
- ◯ 7 days

On average, HOW LONG (in minutes) did you spend PER DAY, during THIS WEEK, doing your AEROBIC EXERCISES?

____

For the times you did the AEROBIC EXERCISES that you were prescribed, how might you describe your exertion (how hard you worked out)?

- ◯ Rest, no feeling of exertion (e.g., this would be the equivalent of reading a book, or watching television)
- ◯ Very, very light (e.g., tying shoes)
- ◯ Very light (e.g., chores like folding clothes that seem to take little effort)
- ◯ Fairly light (e.g., walking through the grocery store or other activities that require some effort but not enough to speed up your breathing)
- ◯ Somewhat hard (e.g., brisk walking or other activities that require moderate effort and speed your heart rate and breathing but don't make you out of breath)
- ◯ Hard (e.g., bicycling, swimming, or other activities that take vigorous effort and get the heart pounding and make breathing very fast)
- ◯ Very hard (e.g., the highest level of activity you can sustain)
- ◯ Very, very hard (e.g., a finishing kick in a race or other burst of activity that you can't maintain for long)

The following questions are about your engagement in STRENGTH EXERCISES:

During the PC-PEP Intervention/Training with our registered Certified Exercise Practitioner Jeff Zahavich, we encouraged you to do strength exercises/training TWO times a week.

HOW MANY DAYS THIS WEEK, did you do the Strength EXERCISES/TRAINING that you were prescribed by the Study's Exercise Expert?

- ◯ not at all
- ◯ 1 day
- ◯ 2 days
- ◯ 3 days
- ◯ 4 days
- ◯ 5 days
- ◯ 6 days
- ◯ 7 days

On average, HOW LONG (in minutes) did you spend PER DAY, during THIS WEEK, doing your STRENGTH EXERCISES?

____

For the times you did the STRENGTH EXERCISES/TRAINING that you were prescribed, how might you describe your exertion (how hard you worked out)?

- ◯ Rest, no feeling of exertion (e.g., this would be the equivalent of reading a book, or watching television)
- ◯ Very, very light (e.g., tying shoes)
- ◯ Very light (e.g., chores like folding clothes that seem to take little effort)
- ◯ Fairly light (e.g., walking through the grocery store or other activities that require some effort but not enough to speed up your breathing)
- ◯ Somewhat hard (e.g., brisk walking or other activities that require moderate effort and speed your heart rate and breathing but don't make you out of breath)
- ◯ Hard (e.g., bicycling, swimming, or other activities that take vigorous effort and get the heart pounding and make breathing very fast)
- ◯ Very hard (e.g., the highest level of activity you can sustain)
- ◯ Very, very hard (e.g., a finishing kick in a race or other burst of activity that you can't maintain for long)

The following questions are about your engagement in PELVIC FLOOR (KEGELS) EXERCISES:

During the PC-PEP Intervention/Training with our Pelvic Floor Nurse, we encouraged you to do pelvic floor (KEGELS) exercises, 3 times a day; for 10 minutes each time.

HOW MANY DAYS THIS WEEK, did you do the PELVIC FLOOR (KEGEL) EXERCISES that you were prescribed by the Study's Physiotherapist/or Pelvic Floor Nurse?

- ◯ not at all
- ◯ 1 day
- ◯ 2 days
- ◯ 3 days
- ◯ 4 days
- ◯ 5 days
- ◯ 6 days
- ◯ 7 days

On average, HOW LONG (in minutes) did you spend PER DAY, during THIS WEEK, doing KEGELS?

____

The following questions are about your engagement in MEDITATION.

During the PC-PEP Intervention/Training, we encouraged you to meditate once a day, for 10 minutes

HOW MANY DAYS THIS WEEK, did you Meditate?

- ◯ not at all
- ◯ 1 day
- ◯ 2 days
- ◯ 3 days
- ◯ 4 days
- ◯ 5 days
- ◯ 6 days
- ◯ 7 days

On average, HOW LONG (in minutes) did you spend PER DAY, during THIS WEEK, meditating?

____

During the PC-PEP Intimacy and Connection Training of the Program we asked you to engage in at least one form of intimacy and connection every day, at least once. The following questions are about your engagement in the PC-PEP Connection and Intimacy exercises we prescribed.

HOW MANY DAYS THIS WEEK, did you engage in EMOTIONAL INTIMACY (e.g., sharing your feelings with another person, being vulnerable and authentic about how you really feel)?

- ◯ not at all
- ◯ 1 day
- ◯ 2 days
- ◯ 3 days
- ◯ 4 days
- ◯ 5 days
- ◯ 6 days
- ◯ 7 days

HOW MANY DAYS THIS WEEK, did you engage in INTELLECTUAL INTIMACY (e.g., exchanging ideas and thoughts, positive and negative, about how you think about things and how you make sense about things you care or think about)?

- ◯ not at all
- ◯ 1 day
- ◯ 2 days
- ◯ 3 days
- ◯ 4 days
- ◯ 5 days
- ◯ 6 days
- ◯ 7 days

HOW MANY DAYS THIS WEEK, did you engage in PHYSICAL INTIMACY (e.g., being in close proximity to another person/or could be a pet; touching; kissing; cuddling; caressing and/or hugging a person or could be a pet; sexual intercourse, etc.)?

- ◯ not at all
- ◯ 1 day
- ◯ 2 days
- ◯ 3 days
- ◯ 4 days
- ◯ 5 days
- ◯ 6 days
- ◯ 7 days

HOW MANY DAYS THIS WEEK, did you engage in RECREATIONAL INTIMACY (sharing in the pleasure of doing an activity together - whether active or non-active, e.g., skating, skiing, playing cards, dancing, golfing, swimming, walking, watching a movie together, hiking, traveling, doing some class together - such as a yoga class or an exercise class; or any other type of recreational activity)?

- ◯ not at all
- ◯ 1 day
- ◯ 2 days
- ◯ 3 days
- ◯ 4 days
- ◯ 5 days
- ◯ 6 days
- ◯ 7 days

HOW MANY DAYS THIS WEEK, did you engage in SELF INTIMACY (e.g., being aware of your own feelings, caring about those feelings, feeling deeply connected with how you feel and think and having love and compassion for yourself)?

- ◯ not at all
- ◯ 1 day
- ◯ 2 days
- ◯ 3 days
- ◯ 4 days
- ◯ 5 days
- ◯ 6 days
- ◯ 7 days

HOW MANY DAYS THIS WEEK, did you engage in OTHER TYPES INTIMACY/ activities where two people are mutually vulnerable, open, mindfully connected while sharing and activity. Examples may include, but not be limited to the following:

Spiritual intimacy - sharing in your spiritual journey with another, having common ethics, qualities and morals.

Unconditional intimacy- sharing in unconditionally loving someone, seeing them as the spirit that lives within rather than their appearance, their social role, their occupation or wealth.

Sleep intimacy- feeling safe and happy to share your private sleeping space with someone.

Aesthetic intimacy - sharing in the pleasure of listening to music, or watching movies, or tasking foods, or viewing works of art in an art galley.

Food intimacy - preparing and eating of the food prepared with others while connecting with one another and sharing in the pleasure of the food's taste.

Technical intimacy - sharing in reaching a common technical goal with another and working together to get there (e.g., fixing a car, a computer, and algorithm, and appliance that is not working).

Financial intimacy - figuring out together with another person how to do well with what resources you have.

- ◯ not at all
- ◯ 1 day
- ◯ 2 days
- ◯ 3 days
- ◯ 4 days
- ◯ 5 days
- ◯ 6 days
- ◯ 7 days

If you answered 1 to 7 days to the previous question please tell us what were some of the OTHER types of INTIMACY that you engaged in, during this past week? ______________________________

How many servings of fruit (1 cup) did you eat EACH DAY, on average, this week?

- ◯ None
- ◯ less than 2 servings
- ◯ 2-4 servings
- ◯ 5 or more servings on average, each day

How many servings of vegetables (fresh or cooked, not fried) did you eat EACH DAY, on average, this week? 1 serving of vegetables is 1 cup.

- ◯ None
- ◯ less than 2 servings
- ◯ 2-4 servings
- ◯ 5 or more servings on average, each day

How many servings (1 serving is the equivalent of 30 grams of mixture of peanuts, Brazilian nuts, walnuts, almonds, or hazelnuts) of nuts did you eat EACH DAY, on average, this week?

- ◯ I did not eat any nuts this week
- ◯ 2 or less
- ◯ 3-4 times
- ◯ 5 or more times on average, each day

How many spoons of extra virgin olive oil did you consume EACH DAY, on average, this week?

- ◯ None
- ◯ 3 or less
- ◯ 4 or more times on average, each day

How many times did you eat beans (like lentils, peas, pinto, or black beans), chicken, white - not red - meat, fish, EACH DAY, on average, this week?

- ◯ None
- ◯ less than 1 time
- ◯ 1-2 times
- ◯ 3 to 5 times
- ◯ more than 5 times on average, each day

How many times did you eat red meat THIS WEEK?

- ◯ I did not eat any red meat this week
- ◯ 1-2 times this week
- ◯ 3-4 times this week
- ◯ 5 times or more this week

How many times did you smoke, THIS WEEK?

- ◯ I did not smoke any cigarettes or other products this week
- ◯ Once
- ◯ 2-3 times
- ◯ 4-6 times
- ◯ More than 6 times

How many glasses of wine or other alcoholic beverages did you consume THIS WEEK?

- ◯ I did not consume any alcohol this week
- ◯ I only consumed wine and did so no more than 3 standard drinks a day, on average
- ◯ I only consumed wine and did so more than 3 standard drinks a day, on average
- ◯ I consumed wine AND/OR other alcoholic beverages and did so no more than 3 standard drinks a day, on average
- ◯ I consumed wine AND/OR other alcoholic beverages and did so more than 3 standard drinks a day, on average

How many times did you eat fast food meals or snacks EACH DAY on average, this week?

- ◯ I did not consume any fast food meals or snacks this week
- ◯ less than 1 time a day
- ◯ 1-2 times a day
- ◯ 3-4 times a day
- ◯ 5 or more times a day

How many regular sodas and glasses of sweet tea did you drink EACH DAY on average, this week?

- ◯ None
- ◯ 1 time or less
- ◯ 2-3 times
- ◯ 4 or more times

How many times did you eat regular snack chips or crackers (not low fat) EACH DAY on average, this week?

- ◯ None
- ◯ 1 time or less
- ◯ 2-3 times
- ◯ 4 or more times

How many times did you eat commercial bakery goods, sweets, or pastries (not low-fat kind) EACH DAY on average, this week?

- ◯ None
- ◯ 1 time or less
- ◯ 2-3 times
- ◯ 4 or more times

How much margarine, butter, or meat fat did you use to season vegetables or put on potatoes, toast, or corn EACH DAY on average, this week?

- ◯ None
- ◯ Very little (half a spoon or less)
- ◯ Some (between half-a-spoon and a spoon-and-a-half)
- ◯ A lot (more than a spoon-and-a-half)

How many times did you eat processed meats EACH DAY on average, this week?

- ◯ None
- ◯ 100 grams or less
- ◯ More than 100 grams each day this week on average

What dietary changes, if any, have you made in your diet THIS WEEK that you think are helpful to you in light of your prostate cancer diagnosis?

How many times did you connect with your Buddy/Buddies and/or Mentor/Mentors THIS WEEK?

- ◯ None
- ◯ Once
- ◯ Two or more times

Table S1. Two-level linear model analysis for the whole sample (A) and by treatment modality (B) fitting the Dyadic Adjustment Scale sum score and its subscales (Affection, Consensus, Satisfaction, Cohesion) among 119 prostate cancer patients evaluating differences between groups (waitlist control vs PC-PEP) from baseline to 6-months, and baseline to 12-months *without covariates adjustment*.

A. Full Sample (n = 119)	**Baseline to 6 months**				**Baseline to 12 months**			
	**DAS Sum score (n=119)**							
Level	*Parameter Estimate*	*95% Confidence Interval*		*p value*	*Parameter Estimate*	*95% Confidence Interval*		*p value*
		Lower	*Upper*			*Lower*	*Upper*	
Group (Control vs. PC-PEP)	-5.7	-13	1.3	0.11	-6.2	-13	0.98	0.090
Time (baseline vs. 6 months)	0.31	-2.6	3.2	0.8	1.3	-3.7	6.2	0.6
Time x Group (PC-PEP)	1.2	-2.9	5.3	0.6	1.6	-5.4	8.6	0.7
	**DAS Consensus score**							
Group (Control vs. PC-PEP)	-1.6	-4.9	1.7	0.3	-1.7	-5.1	1.7	0.3
Time (baseline vs. 6 months)	1.3	-0.74	3.2	0.2	1.6	-1.2	4.3	0.3
Time x Group (PC-PEP)	-0.35	-3.1	2.4	0.8	-0.30	-4.1	3.6	0.9
	**DAS Affection score**							
Group (Control vs. PC-PEP)	-0.50	-1.5	0.45	0.3	-0.31	-1.2	0.60	0.5
Time (baseline vs. 6 months)	0.11	-0.39	0.61	0.7	0.29	-0.34	0.92	0.4
Time x Group (PC-PEP)	0.11	-0.59	0.81	0.8	-0.063	-0.95	0.82	0.9
	**DAS Satisfaction score**							
Group (Control vs. PC-PEP)	-2.2	-4.5	0.020	0.052	-2.7	-5.0	-0.37	0.023
Time (baseline vs. 6 months)	-0.26	-1.5	1.0	0.7	0.43	-1.1	2.0	0.6
Time x Group (PC-PEP)	0.56	-1.2	2.3	0.5	1.0	-1.2	3.2	0.4
	**DAS Cohesion score**							
Group (Control vs. PC-PEP)	-1.2	-2.8	0.43	0.15	-1.5	-3.1	0.13	0.07
Time (baseline vs. 6 months)	-0.74	-1.5	0.042	0.064	-1.0	-2.1	0.027	0.056
Time x Group (PC-PEP)	0.69	-0.41	1.8	0.2	0.96	-0.53	2.5	0.2

B. Radical Prostatectomy (n= 57)	**Baseline to 6 months**				**Baseline to 12 months**			
	**DAS Sum score**							
Level	*Parameter Estimate*	*95% Confidence Interval*		*p value*	*Parameter Estimate*	*95% Confidence Interval*		*p value*
		Lower	*Upper*			*Lower*	*Upper*	
Group (Control vs. PC-PEP)	-16	-26	-6.2	0.002	-16	-26	-6.0	0.5
Time (baseline vs. 6 months)	-2.2	-7.1	2.7	0.4	-2.5	-10	4.9	0.5
Time x Group (PC-PEP)	6.0	-0.61	13	0.074	6.0	-4.0	16	0.2
	**DAS Consensus score**							
Group (Control vs. PC-PEP)	-5.6	-9.5	-1.7	0.005	-6.5	-11	-2.2	0.003
Time (baseline vs. 6 months)	-0.49	-2.7	1.7	0.7	-1.3	-4.8	2.3	0.5
Time x Group (PC-PEP)	1.9	-1.1	4.8	0.2	2.7	-2.0	7.5	0.3
	**DAS Affection score**							
Group (Control vs. PC-PEP)	-1.8	-3.1	-0.45	0.009	-1.5	-2.7	-0.29	0.015
Time (baseline vs. 6 months)	-0.18	-0.96	0.61	0.7	-0.16	-1.1	0.77	0.7
Time x Group (PC-PEP)	0.71	-0.34	1.8	0.18	0.48	-0.78	1.7	0.5
	**DAS Satisfaction score**							

Group (Control vs. PC-PEP)	-5.2	-8.6	-1.9	0.003	-4.9	-8.4	-1.4	0.007
Time (baseline vs. 6 months)	-0.70	-2.9	1.5	0.5	-0.21	-2.9	2.5	0.9
Time x Group (PC-PEP)	2.1	-0.81	5.0	0.15	1.7	-1.9	5.3	0.4
	**DAS Cohesion score**							
Group (Control vs. PC-PEP)	-3.2	-5.6	-0.85	0.009	-3.2	-5.5	-0.78	0.010
Time (baseline vs. 6 months)	-0.81	-1.9	0.28	0.14	-0.87	-2.3	0.54	0.2
Time x Group (PC-PEP)	1.2	-0.27	2.7	0.11	0.98	-0.92	2.9	0.3
C. Radiation Therapy (n= 61)	**Baseline to 6 months**				**Baseline to 12 months**			
	**DAS Sum score**							
Group (Control vs. PC-PEP)	4.4	-5.7	14	0.4	2.8	-7.4	13	0.6
Time (baseline vs. 6 months)	2.3	-1.1	5.7	0.18	4.2	-2.7	11	0.2
Time x Group (PC-PEP)	-3.5	-8.5	1.6	0.17	-1.9	-12	8.2	0.7
	**DAS Consensus score**							
Group (Control vs. PC-PEP)	2.1	-3.2	7.4	0.4	2.6	-2.6	7.7	0.3
Time (baseline vs. 6 months)	2.6	-0.55	5.8	0.10	3.8	-0.29	7.9	0.068
Time x Group (PC-PEP)	-2.2	-6.9	2.5	0.4	-2.7	-8.7	3.4	0.4
	**DAS Affection score**							
Group (Control vs. PC-PEP)	0.75	-0.64	2.1	0.3	0.80	-0.56	2.2	0.3
Time (baseline vs. 6 months)	0.33	-0.32	0.99	0.3	0.64	-0.24	1.5	0.2
Time x Group (PC-PEP)	-0.48	-1.4	0.48	0.3	-0.53	-1.8	0.76	0.4
	**DAS Satisfaction score**							
Group (Control vs. PC-PEP)	0.79	-2.2	3.8	0.6	-0.63	-3.7	2.5	0.7
Time (baseline vs. 6 months)	0.11	-1.3	1.5	0.9	0.94	-0.96	2.8	0.3
Time x Group (PC-PEP)	-1.1	-3.2	1.0	0.3	0.35	-2.5	3.2	0.8
	**DAS Cohesion score**							
Group (Control vs. PC-PEP)	0.82	-1.4	3.0	0.5	0.062	-2.2	2.3	1.0
Time (baseline vs. 6 months)	-0.68	-1.8	0.46	0.2	-1.2	-2.7	0.44	0.2
Time x Group (PC-PEP)	0.18	-1.5	1.9	0.8	0.94	-1.4	3.3	0.4

Table S2. Two-level linear model analysis for the whole sample (A) and by treatment modality (B) fitting the Functional Assessment of Cancer Therapy – Prostate (FACT-P) and the Functional Assessment of Chronic Illness Therapy – Spiritual Wellbeing (FACIT-Sp-12) among 119 prostate cancer patients evaluating differences between groups (waitlist control vs PC-PEP from baseline to 6 months, and baseline to 12 months, *without covariates adjustment.*

A. Full Sample (n = 119)	**Baseline to 6 months**				**Baseline to 12 months**			
	**FACT-P Sum score (n=119)**							
Level	*Parameter Estimate*	*95% Confidence Interval*		*p value*	*Parameter Estimate*	*95% Confidence Interval*		*p value*
		Lower	*Upper*			*Lower*	*Upper*	
Group (Control vs. PC-PEP)	-3.8	-8.2	0.63	0.093	-5.4	-9.9	-0.82	0.021
Time (baseline vs. 6 months)	-2.3	-4.9	0.34	0.088	-1.6	-5.0	1.7	0.3
Time x Group (PC-PEP)	1.8	-1.9	5.4	0.34	3.4	-1.3	8.1	0.15
	**FACT-P Social Wellbeing score**							
Group (Control vs. PC-PEP)	-1.5	-3.3	0.42	0.13	-1.3	-3.2	0.69	0.2
Time (baseline vs. 6 months)	0.47	-0.60	1.5	0.39	1.0	-0.41	2.4	0.16
Time x Group (PCPEP)	-0.20	-1.7	1.3	0.79	-0.38	-2.4	1.6	0.7
	**FACT-P Emotional Wellbeing score**							
Group (Control vs. PC-PEP)	-1.1	-2.5	0.27	0.11	-1.5	-3.0	-0.066	0.041
Time (baseline vs. 6 months)	-2.2	-3.4	-1.1	<0.001	-2.2	-3.4	-1.0	<0.001
Time x Group (PC-PEP)	1.3	-0.27	3.0	0.10	1.7	0.10	3.4	0.037
	**FACT-P Functional Wellbeing score**							
Group (Control vs. PC-PEP)	-1.2	-3.2	0.75	0.23	-2.6	-4.6	-0.60	0.011
Time (baseline vs. 6 months)	-0.50	-1.7	0.71	0.4	-0.46	-1.9	1.0	0.5
Time x Group (PC-PEP)	0.61	-1.1	2.3	0.5	2.0	-0.023	4.1	0.053
	**FACIT-Sp-12 Sum score**							
Group (Control vs. PC-PEP)	-3.3	-6.5	-0.042	0.047	-3.7	-7.1	-0.35	0.031
Time (baseline vs. 6 months)	-2.1	-3.9	-0.34	0.02	-1.4	-3.9	1.1	0.3
Time x Group (PC-PEP)	1.7	-0.79	4.2	0.18	2.2	-1.3	5.7	0.2
	**FACIT-Sp-12 Meaning score**							
Group (Control vs. PC-PEP)	-0.30	-1.4	0.76	0.6	-0.90	-2.1	0.28	0.13
Time (baseline vs. 6 months)	0.20	-0.50	0.90	0.6	0.66	-0.29	1.6	0.17
Time x Group (PC-PEP)	0.097	-0.88	1.1	0.9	0.71	-0.62	2.0	0.3
	**FACIT-Sp-12 Peace score**							
Group (Control vs. PC-PEP)	-1.0	-2.2	0.26	0.12	-2.0	-3.2	-0.70	0.002
Time (baseline vs. 6 months)	-0.70	-1.4	0.031	0.060	-0.74	-1.6	0.13	0.094
Time x Group (PC-PEP)	0.32	-0.71	1.3	0.5	1.3	0.076	2.5	0.037
	**FACIT-Sp-12 Faith score**							
Group (Control vs. PC-PEP)	-2.0	-3.7	-0.29	0.022	-0.86	-2.6	0.84	0.3
Time (baseline vs. 6 months)	-1.6	-2.6	-0.70	<0.001	-1.3	-2.5	-0.091	0.035
Time x Group (PC-PEP)	1.3	-0.00084	2.6	0.050	0.18	-1.5	1.9	0.8

B. Radical Prostatectomy (n = 57)	**Baseline to 6 months**				**Baseline to 12 months**			
	**FACT-P Sum score**							
Level		*95% Confidence Interval*		*p value*		*95% Confidence Interval*		*p value*

	Parameter Estimate	*Lower*	*Upper*		*Parameter Estimate*	*Lower*	*Upper*	
Group (Control vs. PC-PEP)	-11	-17	-4.9	<0.001	-8.0	-14	-2.2	0.008
Time (baseline vs. 6 months)	-2.3	-6.5	2.0	0.29	1.4	-5.5	2.6	0.5
Time x Group (PC-PEP)	4.6	-1.0	10	0.11	1.8	-3.7	7.2	0.5
	**FACT-P Social Wellbeing score**							
Group (Control vs. PC-PEP)	-4.1	-6.6	-1.6	0.002	-2.5	-5.0	-0.0056	0.050
Time (baseline vs. 6 months)	0.61	-0.95	2.2	0.4	1.4	-0.35	3.1	0.12
Time x Group (PCPEP)	0.71	-1.4	2.8	0.5	-0.82	-3.1	1.5	0.5
	**FACT-P Emotional Wellbeing score**							
Group (Control vs. PC-PEP)	-2.5	-4.7	-0.30	0.026	-1.6	-3.6	0.36	0.11
Time (baseline vs. 6 months)	-1.8	-3.7	0.10	0.063	-2.0	-3.5	-0.50	0.010
Time x Group (PC-PEP)	1.7	-0.81	4.3	0.18	0.84	-1.2	2.8	0.4
	**FACT-P Functional Wellbeing score**							
Group (Control vs. PC-PEP)	-4.4	-7.0	-1.7	0.001	-3.9	-6.5	-1.3	0.004
Time (baseline vs. 6 months)	-1.1	-3.1	0.99	0.3	-0.79	-2.7	1.1	0.4
Time x Group (PC-PEP)	2.2	-0.56	5.0	0.12	1.7	-0.85	4.3	0.19
	**FACIT-Sp-12 Sum score**							
Group (Control vs. PC-PEP)	-6.7	-11	-2.2	0.004	-5.8	-10	-1.1	0.015
Time (baseline vs. 6 months)	-3.0	-5.5	-0.53	0.018	-2.2	-5.3	0.92	0.16
Time x Group (PC-PEP)	3.4	0.030	6.7	0.048	2.5	-1.7	6.6	0.2
	**FACIT-Sp-12 Meaning score**							
Group (Control vs. PC-PEP)	-1.5	-3.0	-0.098	0.036	-1.2	-2.7	0.35	0.13
Time (baseline vs. 6 months)	-0.15	-1.2	0.85	0.8	0.60	-0.51	1.7	0.3
Time x Group (PC-PEP)	0.90	-0.44	2.3	0.19	0.53	-0.96	2.0	0.5
	**FACIT-Sp-12 Peace score**							
Group (Control vs. PC-PEP)	-2.6	-4.4	-0.92	0.003	-2.9	-4.7	-1.2	0.001
Time (baseline vs. 6 months)	-1.0	-2.1	0.14	0.086	-0.95	-1.9	0.028	0.057
Time x Group (PC-PEP)	1.1	-0.40	2.6	0.15	1.4	0.048	2.7	0.042
	**FACIT-Sp-12 Faith score**							
Group (Control vs. PC-PEP)	-2.5	-4.8	-0.23	0.031	-1.7	-4.0	0.64	0.15
Time (baseline vs. 6 months)	-1.9	-3.3	-0.57	0.006	-1.8	-3.7	0.021	0.053
Time x Group (PC-PEP)	1.4	-0.42	3.2	0.13	0.56	-1.9	3.1	0.7
D. Radiation Therapy (n=61)	**Baseline to 6 months**				**Baseline to 12 months**			
	**FACT-P Sum score**							
Group (Control vs. PC-PEP)	3.6	-2.7	9.8	0.26	-3.1	-10	3.9	0.4
Time (baseline vs. 6 months)	-2.2	-5.4	0.90	0.16	-1.8	-7.0	3.5	0.5
Time x Group (PC-PEP)	-1.5	-6.2	3.1	0.5	5.2	-2.6	13	0.19
	**FACT-P Social Wellbeing score**							
Group (Control vs. PC-PEP)	1.3	-1.4	4.0	0.3	0.022	-3.0	3.0	1.0
Time (baseline vs. 6 months)	0.36	-1.1	1.8	0.6	0.76	-1.5	3.0	0.5
Time x Group (PCPEP)	-1.3	-3.4	0.84	0.2	-0.0076	-3.3	3.3	1.0
	**FACT-P Emotional Wellbeing score**							

Group (Control vs. PC-PEP)	0.36	-1.4	2.1	0.7	-1.6	-3.7	0.58	0.15
Time (baseline vs. 6 months)	-2.6	-4.0	-1.1	<0.001	-2.3	-4.1	-0.58	0.010
Time x Group (PC-PEP)	0.72	-1.4	2.8	0.5	2.7	0.059	5.3	0.045
	**FACT-P Functional Wellbeing score**							
Group (Control vs. PC-PEP)	1.8	-0.92	4.7	0.2	-1.6	-4.6	1.4	0.3
Time (baseline vs. 6 months)	-0.06	-1.5	1.4	0.9	-0.18	-2.4	2.0	0.9
Time x Group (PC-PEP)	-0.98	-3.1	1.1	0.4	2.5	-0.78	5.8	0.13
	**FACIT-Sp-12 Sum score**							
Group (Control vs. PC-PEP)	0.056	-4.6	4.7	1.0	-2.0	-7.0	3.0	0.4
Time (baseline vs. 6 months)	-1.4	-4.0	1.2	0.3	-0.67	-4.6	3.2	0.7
Time x Group (PC-PEP)	0.11	-3.7	3.9	1.0	2.1	-3.6	7.9	0.5
	**FACIT-Sp-12 Meaning score**							
Group (Control vs. PC-PEP)	0.78	-0.82	2.4	0.3	-0.84	-2.7	0.99	0.4
Time (baseline vs. 6 months)	0.49	-0.50	1.5	0.3	0.73	-0.78	2.2	0.3
Time x Group (PC-PEP)	-0.70	-2.2	0.76	0.3	0.92	-1.3	3.1	0.4
	**FACIT-Sp-12 Peace score**							
Group (Control vs. PC-PEP)	0.71	-1.1	2.5	0.4	-1.0	-2.9	0.82	0.3
Time (baseline vs. 6 months)	-0.49	-1.5	0.49	0.3	-0.55	-1.9	0.85	0.4
Time x Group (PC-PEP)	-0.48	-1.9	0.96	0.5	1.3	-0.79	3.3	0.2
	**FACIT-Sp-12 Faith score**							
Group (Control vs. PC-PEP)	-1.4	-4.0	1.1	0.3	-0.10	-2.7	2.5	0.9
Time (baseline vs. 6 months)	-1.4	-2.7	-0.098	0.035	-0.85	-2.4	0.73	0.3
Time x Group (PC-PEP)	1.3	-0.63	3.2	0.18	-0.044	-2.4	2.3	1.0

Supplementary Table – Observed means, standard errors and counts for the **Intimacy and Connection practice over the 26 weeks of the trial** among the 128 participants in the PC-PEP Trial from Halifax, Nova Scotia.

EARLY GROUP

Week number		1			2			3			4			5			6			7			8			9			10			11			12			13		
		M	SE	n	M	SE	n	M	SE	n	M	SE	n	M	SE	n	M	SE	n	M	SE	n	M	SE	n	M	SE	n	M	SE	n	M	SE	n	M	SE	n	M	SE	n
Emotional	Nr. of days/week	4.06	0.30	66	4.36	0.30	66	4.55	0.28	66	4.08	0.31	66	4.50	0.29	66	4.37	0.29	65	4.58	0.30	65	4.58	0.30	65	4.15	0.29	66	4.27	0.29	66	4.28	0.31	65	4.71	0.30	66	4.58	0.28	64
Intellectual	Nr. of days/week	4.03	0.29	66	4.32	0.31	66	4.45	0.29	66	4.35	0.28	66	4.44	0.29	66	4.43	0.28	65	4.28	0.29	65	4.28	0.29	65	4.33	0.27	66	4.30	0.29	66	4.35	0.31	65	4.59	0.28	66	4.52	0.28	64
Physical	Nr. of days/week	4.65	0.29	66	4.91	0.31	66	4.79	0.31	66	4.94	0.31	66	4.88	0.31	66	4.72	0.32	65	4.88	0.33	65	4.88	0.33	65	4.76	0.30	66	5.02	0.30	66	4.80	0.33	65	5.06	0.31	66	5.06	0.31	64
Recreation	Nr. of days/week	3.95	0.29	66	4.39	0.31	66	4.21	0.30	66	4.14	0.32	66	4.20	0.31	66	4.06	0.32	65	4.34	0.33	65	4.34	0.33	65	4.33	0.30	66	4.62	0.27	66	4.69	0.29	65	4.58	0.30	66	4.77	0.30	64
Self	Nr. of days/week	3.61	0.30	66	3.70	0.32	66	3.71	0.30	66	3.64	0.31	66	3.61	0.30	66	3.57	0.31	65	3.69	0.31	65	3.69	0.31	65	3.50	0.32	66	3.83	0.31	66	3.69	0.32	65	3.92	0.30	66	3.80	0.33	64
Other	Nr. of days/week	4.48	0.32	66	5.17	0.30	66	5.08	0.31	66	5.02	0.30	66	4.94	0.29	66	4.68	0.32	65	4.72	0.32	65	4.72	0.32	65	4.65	0.36	66	4.65	0.33	66	4.69	0.34	65	4.70	0.34	66	4.67	0.35	64

14			15			16			17			18			19			20			21			22			23			24			25			26		
M	SE	n	M	SE	n	M	SE	n	M	SE	n	M	SE	n	M	SE	n	M	SE	n	M	SE	n	M	SE	n	M	SE	n	M	SE	n	M	SE	n	M	SE	n
4.48	0.31	65	4.20	0.31	65	4.30	0.30	64	4.08	0.32	65	4.20	0.29	65	4.14	0.30	64	4.37	0.30	65	4.27	0.29	64	3.91	0.32	65	3.88	0.30	64	3.94	0.31	66	4.11	0.30	65	3.98	0.32	66
4.63	0.30	65	4.34	0.29	65	4.39	0.29	64	4.49	0.29	65	4.54	0.28	65	4.48	0.28	64	4.66	0.28	65	4.52	0.28	64	4.26	0.30	65	3.91	0.30	64	4.27	0.27	66	4.34	0.29	65	4.30	0.30	66
5.11	0.31	65	5.25	0.28	65	5.17	0.30	64	5.20	0.31	65	5.15	0.30	65	5.19	0.31	64	5.22	0.28	65	5.17	0.29	64	4.97	0.33	65	4.84	0.31	64	5.00	0.32	66	5.09	0.30	65	5.23	0.28	66
4.65	0.31	65	4.54	0.29	65	4.52	0.30	64	4.40	0.32	65	4.42	0.30	65	4.42	0.29	64	4.43	0.29	65	4.63	0.31	64	4.48	0.31	65	4.53	0.27	64	4.62	0.29	66	4.57	0.30	65	4.58	0.29	66
3.82	0.33	65	3.62	0.31	65	3.97	0.33	64	3.92	0.32	65	3.71	0.33	65	3.47	0.34	64	3.72	0.32	65	3.53	0.33	64	3.42	0.33	65	3.28	0.31	64	3.56	0.32	66	3.72	0.32	65	3.58	0.31	66
4.95	0.34	65	4.83	0.34	65	4.70	0.35	64	4.72	0.35	65	4.66	0.34	65	4.77	0.35	64	4.75	0.35	65	4.70	0.35	64	4.62	0.35	65	4.72	0.33	64	4.83	0.34	66	4.54	0.35	65	4.53	0.35	66

LATE GROUP

Week number		1			2			3			4			5			6			7			8			9			10			11			12			13		
		M	SE	n	M	SE	n	M	SE	n	M	SE	n	M	SE	n	M	SE	n	M	SE	n	M	SE	n	M	SE	n	M	SE	n	M	SE	n	M	SE	n	M	SE	n
Emotional	Nr. of days/week	3.46	0.35	48	3.76	0.37	45	3.93	0.34	43	3.79	0.37	43	3.51	0.35	43	3.53	0.35	43	3.57	0.35	42	3.57	0.35	42	3.65	0.38	40	3.92	0.40	38	3.74	0.37	39	3.60	0.37	40	3.45	0.35	40
Intellectual	Nr. of days/week	3.88	0.33	48	3.96	0.32	45	3.81	0.29	43	3.91	0.36	43	3.63	0.35	43	3.74	0.35	43	3.52	0.34	42	3.52	0.34	42	3.75	0.38	40	3.97	0.37	38	3.59	0.36	39	3.83	0.34	40	3.73	0.38	40
Physical	Nr. of days/week	4.19	0.37	48	4.44	0.35	45	4.33	0.37	43	4.23	0.38	43	4.21	0.39	43	4.28	0.40	43	4.12	0.41	42	4.12	0.41	42	4.58	0.39	40	4.47	0.39	38	4.69	0.41	39	4.40	0.43	40	4.50	0.45	40
Recreation	Nr. of days/week	3.60	0.36	48	3.82	0.35	45	3.84	0.34	43	3.93	0.39	43	3.95	0.38	43	3.98	0.41	43	4.19	0.34	42	4.19	0.34	42	3.83	0.42	40	4.47	0.39	38	4.44	0.39	39	4.08	0.42	40	4.18	0.40	40
Self	Nr. of days/week	3.33	0.39	48	3.78	0.40	45	3.47	0.36	43	3.19	0.39	43	3.35	0.41	43	3.14	0.37	43	2.88	0.39	42	2.88	0.39	42	2.88	0.39	40	3.00	0.40	38	2.92	0.40	39	3.10	0.38	40	2.45	0.40	40
Other	Nr. of days/week	4.04	0.42	48	4.56	0.37	45	4.07	0.40	43	4.26	0.40	43	4.47	0.44	43	4.60	0.41	43	4.45	0.43	42	4.45	0.43	42	4.47	0.45	40	4.29	0.46	38	4.56	0.44	39	4.53	0.41	40	4.40	0.45	40

14			15			16			17			18			19			20			21			22			23			24			25			26		
M	SE	n	M	SE	n	M	SE	n	M	SE	n	M	SE	n	M	SE	n	M	SE	n	M	SE	n	M	SE	n	M	SE	n	M	SE	n	M	SE	n	M	SE	n
3.72	0.37	39	3.36	0.41	39	3.42	0.39	38	3.50	0.38	38	3.74	0.40	38	3.47	0.42	38	3.84	0.39	38	3.76	0.40	37	3.54	0.41	37	3.51	0.42	37	3.35	0.40	37	3.46	0.41	37	3.33	0.42	36
4.23	0.38	39	3.79	0.39	39	3.55	0.40	38	4.13	0.37	38	4.16	0.38	38	3.95	0.40	38	4.18	0.38	38	4.08	0.37	37	3.65	0.39	37	3.95	0.42	37	3.57	0.41	37	3.51	0.42	37	3.58	0.43	36
4.31	0.42	39	4.10	0.44	39	4.00	0.46	38	4.50	0.42	38	4.63	0.40	38	4.58	0.44	38	4.79	0.42	38	4.30	0.41	37	4.32	0.43	37	4.68	0.44	37	4.00	0.44	37	4.27	0.46	37	4.56	0.46	36
4.38	0.39	39	3.97	0.45	39	4.34	0.44	38	4.32	0.43	38	4.68	0.39	38	4.47	0.44	38	4.45	0.41	38	4.32	0.39	37	4.16	0.40	37	4.81	0.40	37	4.03	0.41	37	4.24	0.41	37	4.31	0.45	36
3.41	0.41	39	2.87	0.39	39	3.08	0.43	38	2.97	0.41	38	3.34	0.40	38	2.87	0.41	38	3.53	0.43	38	3.35	0.41	37	2.86	0.41	37	3.22	0.44	37	2.81	0.41	37	3.08	0.44	37	2.86	0.44	36
4.26	0.46	39	4.33	0.45	39	4.21	0.47	38	4.08	0.44	38	4.63	0.46	38	4.47	0.47	38	4.32	0.45	38	4.46	0.44	37	4.24	0.47	37	4.38	0.49	37	4.30	0.47	37	4.46	0.46	37	4.72	0.48	36
